# Protein Translation Inhibition is Involved in the Activity of the Pan-PIM Kinase Inhibitor PIM447 in Combination with Pomalidomide-Dexamethasone in Multiple Myeloma

**DOI:** 10.3390/cancers12102743

**Published:** 2020-09-24

**Authors:** Teresa Paíno, Lorena González-Méndez, Laura San-Segundo, Luis A. Corchete, Susana Hernández-García, Andrea Díaz-Tejedor, Esperanza M. Algarín, Pedro Mogollón, Montserrat Martín-Sánchez, Norma C. Gutiérrez, María-Victoria Mateos, Mercedes Garayoa, Enrique M. Ocio

**Affiliations:** 1 Centro de Investigación del Cáncer-IBMCC (CSIC-Universidad de Salamanca), Complejo Asistencial Universitario de Salamanca-IBSAL, 37007 Salamanca, Spain; lgonzalez@usal.es (L.G.-M.); duckey@usal.es (L.S.-S.); lacorsan@usal.es (L.A.C.); suherga@usal.es (S.H.-G.); adiaz062@usal.es (A.D.-T.); macalgpac@usal.es (E.M.A.); pmog@usal.es (P.M.); monseratt@usal.es (M.M.-S.); normagu@usal.es (N.C.G.); mvmateos@usal.es (M.-V.M.); mgarayoa@usal.es (M.G.); 2 Centro de Investigación Biomédica en Red de Cáncer (CIBERONC, CB16/12/00233), Instituto de Salud Carlos III, 37007 Salamanca, Spain; 3 Hematology Department, Hospital Universitario Marqués de Valdecilla (IDIVAL). Universidad de Cantabria, 39008 Santander, Spain

**Keywords:** multiple myeloma, pan-PIM kinase inhibitor, drug combination, protein translation

## Abstract

**Simple Summary:**

The identification of new pharmacological combinations with synergistic effect in multiple myeloma is highly relevant since this is, for the moment, an incurable disease. The high expression of Proviral Insertion site for Moloney murine leukemia virus (PIM) kinases, especially PIM2, in myeloma cells is in agreement with the antiproliferative effect previously shown by the pan-PIM kinase inhibitor PIM447 in these types of cells. Therefore, PIM447 is a good candidate to be studied in new combinations with standard-of-care drugs. In this work, we demonstrate by preclinical studies that PIM447 in combination with the standard treatment pomalidomide + dexamethasone exerts a potent antitumor effect and significantly improves survival with respect to the standard treatment. Our data suggest that these effects are in part mediated by the inhibition of protein translation promoted by this triple combination. These results could be the basis for new clinical trials based on this all-oral combination, which would benefit MM patients.

**Abstract:**

Background: Proviral Insertion site for Moloney murine leukemia virus (PIM) kinases are overexpressed in hematologic malignancies, including multiple myeloma. Previous preclinical data from our group demonstrated the anti-myeloma effect of the pan-PIM kinase inhibitor PIM447. Methods: Based on those data, we evaluate here, by in vitro and in vivo studies, the activity of the triple combination of PIM447 + pomalidomide + dexamethasone (PIM-Pd) in multiple myeloma. Results: Our results show that the PIM-Pd combination exerts a potent anti-myeloma effect in vitro and in vivo, where it markedly delays tumor growth and prolongs survival of treated mice. Mechanism of action studies performed in vitro and on mice tumor samples suggest that the combination PIM-Pd inhibits protein translation processes through the convergent inhibition of c-Myc and mTORC1, which subsequently disrupts the function of eIF4E. Interestingly the MM pro-survival factor IRF4 is also downregulated after PIM-Pd treatment. As a whole, all these molecular changes would promote cell cycle arrest and deregulation of metabolic pathways, including glycolysis and lipid biosynthesis, leading to inhibition of myeloma cell proliferation. Conclusions: Altogether, our data support the clinical evaluation of the triple combination PIM-Pd for the treatment of patients with multiple myeloma.

## 1. Introduction

The Proviral Insertion site for Moloney murine leukemia virus (PIM) family of serine/threonine kinase proteins is composed of three isoforms (PIM1, PIM2, and PIM3) with high homology and functional redundancy [1]. PIM kinases are widely expressed in cancer with particularly higher expression in hematologic tumors [2], making them especially sensitive to PIM inhibitors [3]. PIM kinases exert their oncogenic effects through the phosphorylation of different proteins mainly involved in cell proliferation and survival (for review, see Blanco-Aparicio et al.; Mondello et al.) [1,3]. For example, PIM kinases have been described to prevent apoptosis by phosphorylating the proapoptotic Bcl-2–associated agonist of cell death (Bad), to be involved in cell cycle regulation through the phosphorylation of the cyclin-dependent kinase inhibitors p21 and p27, and to increase protein synthesis by the phosphorylation of the translational repressor 4E-BP1 [1,3]. In multiple myeloma (MM), PIM2 expression in particular has been shown to be higher than in other hematologic malignancies [2]. Interestingly, different cellular and soluble components of the bone marrow microenvironment cooperatively enhance PIM2 expression in MM cells, thus promoting antiapoptotic effects [4], and PIM2 is also required for maintaining myeloma cell growth through modulating TSC2 phosphorylation, a negative regulator of mTORC1 [5]. 

Several PIM inhibitors have been preclinically evaluated in MM with promising activity [6,7,8]. Among them, our group recently showed the direct anti-myeloma effect of the pan-PIM kinase inhibitor PIM447 (formerly, LGH447) as monotherapy, based on cell-cycle disruption and apoptosis induction, together with a bone-protective effect [6]. Moreover, in the same work, we demonstrated by in vitro studies a very strong synergism of PIM447 with standard-of-care anti-myeloma treatments, such as bortezomib and immunomodulatory agents (lenalidomide and pomalidomide) combined with dexamethasone [6], although the mechanism of action of these combinations was not yet explored.

It is important to mention that PIM447 was the first drug of its class to be evaluated in monotherapy in a phase I clinical trial for relapsed and/or refractory MM patients, demonstrating single-agent antitumor activity and a tolerable safety profile [9]. Nevertheless, MM therapy relies on the use of pharmacological combinations, rather than drugs alone, which is particularly relevant for agents with a mechanism of action relying on a single target. In addition to the synergy of PIM447 with standard anti-myeloma agents reported by our group [6], other synergistic combinations of PIM inhibitors with either classical chemotherapeutic or novel agents have been reported in preclinical models of several hematologic diseases [10,11,12], including MM [7,8,13]. More specifically, the synergism of the pan-PIM kinase inhibitor INCB053914 with itacitinib (JAK1-selective inhibitor) and the decrease of MYC levels induced by this combination in MM have been described [8]. Also, the pan-PIM kinase inhibitor SGI1776 enhances lenalidomide’s anti-myeloma activity due to more effective degradation of IKZF1 and IKZF3 in MM cell lines as well as xenografts of myeloma tumors [13]. In addition, the PIM2-selective inhibitor JP11646 sensitized MM cells to the standard agents melphalan, dexamethasone, and bortezomib [7]. All these data suggest the potential beneficial effect of PIM inhibitor-based combinations for MM patients. Specifically, the ongoing clinical development of PIM447 [9] makes combinations based on this pan-PIM kinase inhibitor especially interesting and highlights the importance of understanding the mechanism of action of such combinations. 

Here, we demonstrate the potent effect of the triple combination of PIM447 + pomalidomide + dexamethasone (PIM-Pd) in delaying tumor growth and prolonging survival in human plasmacytoma murine models. Moreover, by the development of in vitro and in vivo studies, we focus our attention on its mechanism of action and suggest that the joint inhibition of mTORC1 and c-Myc exerted by this combination induces a synergistic inhibition of protein synthesis that disrupts cell cycle control and energy metabolism pathways, leading to apoptosis. Altogether, our results support the clinical development of the all-oral combination of PIM447 with pomalidomide and dexamethasone for MM patients.

## 2. Results

### 2.1. The Triple Combination of PIM447 + Pomalidomide + Dexamethasone (PIM-Pd) is Synergistic in Vitro and Overcomes the Protective Effect Conferred by BM-MSCs

**Figure 1:**
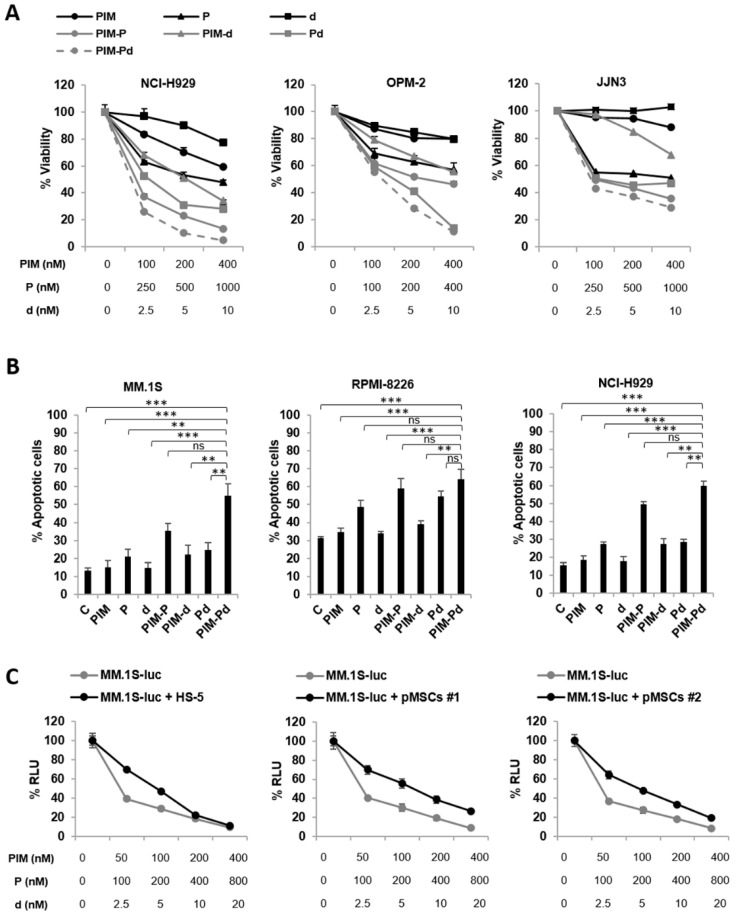
The triple combination PIM-Pd has a synergistic effect on MM cell lines and overcomes the proliferative advantage conferred by BM-MSCs. (**A**) NCI-H929, OPM-2 and JJN3 cell lines were treated for 72 h with the indicated doses of PIM447 (PIM), pomalidomide (P) or dexamethasone (d) alone or in double and triple combinations, and the percentage of cell viability was calculated based on MTT assay (control, 100%). Data represent the mean ± SD. (**B**) MM.1S, RPMI-8226, and NCI-H929 cells were incubated in the absence (control, **C**) or presence of the indicated treatments for 72 h, and the percentage of apoptotic cells was analyzed by flow cytometry after staining with Annexin V-FITC/PI. Data represent the mean ± SEM of three independent experiments. Statistically significant differences among different conditions were evaluated by one-way ANOVA followed by Tukey’s HSD post-hoc test. Differences between PIM-Pd treatment and all other conditions are indicated: ** *p* < 0.01; *** *p* < 0.001; ns = not significant. Doses of PIM/P/d (in nM): 100/200/5 for MM.1S; 100/250/2.5 for RPMI-8226; 200/500/5 for NCI-H929. (**C**) MM.1S-luc cells were co-cultured for 72 h with the HS-5 cell line or with BM-MSCs obtained from two patients with MM in the presence of the indicated dose combinations of PIM-Pd. After the co-culture period, MM.1S-luc viability was assessed by luciferase bioluminescence measurement. Graphs illustrate the mean ± SD.

We recently demonstrated that the PIM-Pd combination shows very strong synergism in vitro in MM.1S and RPMI-8226 cells [6]. Here, the potency of this combination was confirmed in additional MM cell lines (NCI-H929, OPM-2, and JJN3) also finding a synergistic effect (combination index (CI) range for NCI-H929: 0.097–0.148; CI range for OPM-2: 0.004–0.261; CI range for JJN3: 0.234-0.579) (Figure 1A and Table S1). The potency of the PIM-Pd combination was also analyzed in a time kinetics study in NCI-H929, OPM-2, JJN3, MM.1S, and RPMI-8226 cell lines. When we focused on median doses for each cell line we found that, in three out of the five cell lines evaluated (NCI-H929, OPM-2 and MM.1S), better CIs were obtained at 72 h, whereas CIs were very similar at 24 and 72 h for RPMI-8226 cells although the effect (reduction of viability) was better at 72 h (Figure S1). These results also translated into significantly (*p* < 0.01) higher percentages of apoptosis with PIM-Pd compared to the combination PIM-d in three representative cell lines (MM.1S, RPMI-8226, and NCI-H929) and also compared to the doublet Pd in MM.1S and NCI-H929 (Figure 1B). We also evaluated the apoptotic effect of the PIM-Pd combination on primary myeloma cells. To do so, bone marrow samples obtained from five patients with MM, either newly-diagnosed (patients #3 and #4) or relapse/refractory (patients #1, #2 and #5), were cultured ex vivo in the absence or presence of the corresponding treatments for 48 h. From the five patients evaluated, only in patient #1 we found a clear increased percentage of apoptotic myeloma cells with the PIM-Pd combination as compared to the rest of treatments. Also, a slight increase in apoptotic myeloma cells was observed in patient #5 with PIM-Pd (Figure S2). It should be noted that both of them were relapse/refractory patients. Overall, in vitro and ex vivo data suggest that the apoptotic effect of the PIM-Pd combination on myeloma cells is moderate and occurs mainly after a relatively long exposure time (72 h) as observed in MM cell lines.

Since mesenchymal stromal cells are essential in the bone marrow microenvironment, we investigated the effect of PIM-Pd on myeloma cells co-cultured with HS-5 cells or BM-MSCs from two patients. Treatment with increasing doses of PIM-Pd clearly reduced MM.1S-luc cell viability in co-culture, with a pattern similar to that of MM.1S-luc cells in monoculture (Figure 1C), without affecting BM-MSC viability (Figure S3).

### 2.2. The Triple Combination PIM-Pd Delays Tumor Growth and Prolongs Survival in Human Plasmacytoma Murine Models

**Figure 2:**
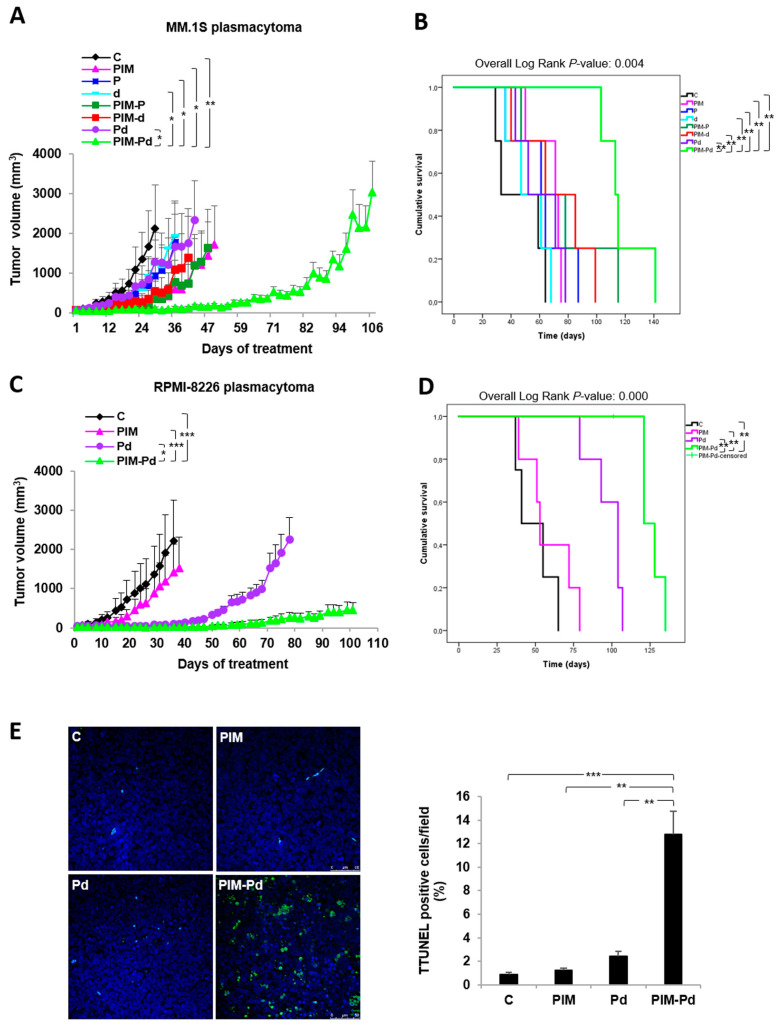
The triple combination PIM-Pd significantly delays tumor growth and improves survival in human plasmacytoma murine models in CB17-SCID mice. (**A**) CB17-SCID mice bearing MM.1S subcutaneous plasmacytomas (generated by injection of 3 × 10^6^ MM.1S cells/mouse) were randomly assigned to receive vehicle (control group; C), PIM447 (PIM), pomalidomide (P), dexamethasone (d), or the respective double and triple combinations (*n* = 4 per group), according to the treatment schedule indicated in the Methods section. Tumor diameters were measured every other day, and tumor volume was estimated as the volume of a 3D ellipse. Data represent the mean ± SEM. Differences in tumor growth among groups were analyzed using the log10 relative tumor volume (RTV) calculated as the final tumor volume, corresponding to the last day for which each curve is represented (when the first mouse of each group died), divided by the initial tumor volume for each mouse. Statistical differences in log10 RTV were assessed by one-way ANOVA followed by Tukey´s HSD post-hoc test. Differences between PIM-Pd treatment and all other conditions are indicated: * *p* < 0.05; ** *p* < 0.01. (**B**), Survival of mice in “A” represented in a Kaplan–Meier curve. Statistical significance was evaluated by the log-rank (Mantel–Cox) test. Differences between PIM-Pd treatment and all other conditions are indicated: ** *p* < 0.01. (**C**), CB17-SCID mice bearing RPMI-8226 subcutaneous plasmacytomas (generated by injection of 5 × 10^6^ RPMI-8226 cells/mouse) were randomly assigned to receive vehicle (control group; C), PIM, Pd, or PIM-Pd (*n* = 5 per group). Treatment schedule and calculation of tumor volume as in “A”. Data represent the mean ± SEM. Statistical analysis was performed as in “A”. Differences between PIM-Pd treatment and all other conditions are indicated: * *p* < 0.05; *** *p* < 0.001. (**D**), Survival of mice in “C” represented in a Kaplan–Meier curve. Statistical significance was evaluated by the log-rank (Mantel–Cox) test. Differences between PIM-Pd treatment and all other conditions are indicated: ** *p* < 0.01. (**E**), Representative micrographs of TUNEL-stained tumor sections from mice treated for two consecutive days with the vehicle (control; C), PIM, Pd or PIM-Pd (scale bar = 50 µm). Bar chart represents the percentage of TUNEL-positive cells of the DAPI-stained total nuclei (12 fields 630× per experimental condition, three plasmacytomas per condition). Data are expressed as the mean ± SEM. Statistically significant differences among treatment groups were evaluated by one-way ANOVA followed by the Games–Howell post-test. Differences between PIM-Pd treatment and all other conditions and are represented as ** *p* < 0.01, *** *p* < 0.001.

Subsequently, the in vivo potency of the combination was evaluated in murine plasmacytomas derived from the MM.1S cell line. PIM-Pd treatment showed a clear tendency to delay tumor growth with respect to double combinations, with statistically significant differences as compared to Pd at each time point studied (*p* < 0.05) and almost reaching statistical significance with respect to PIM-P on day 47 (*p* = 0.0508) (Figure 2A). In fact, the median time to reach an approximated volume of 1000 mm^3^ was doubled in the triple combination with respect to PIM-P, the most potent of the doublets (85 vs. 43 d). All these effects led to a significant increase in the median survival of mice treated with the triple combination (113 d; range: 103–141 d) with respect to PIM-d (64 d; range: 40–99 d; *p* = 0.007) and Pd (52 d; range: 43–78 d; *p* = 0.007), and the tendency, although not significant, was similar with respect to PIM-P (64 d; range: 47–115 d; *p* = 0.153) (Figure 2B). 

The role of PIM447 in potentiating the standard-of-care Pd was confirmed in a RPMI-8226 plasmacytoma model. Accordingly, PIM-Pd treatment significantly delayed tumor growth at each time point studied (*p* < 0.05) and improved median survival with respect to Pd treatment ((PIM-Pd: 121 d (range: 101–135); Pd: 104 d (range: 79–107); *p* = 0.004)) (Figure 2C,D). 

PIM-Pd treatment was generally well tolerated with less than 15% body weight loss in either of the two models (Figure S4). Moreover, when we monitored several potential toxicity/distress symptoms during the course of the experiment, we only observed changes in fur after PIM-Pd treatment, something also observed in some mice treated with the standard Pd (Tables S2 and S3). It should be mentioned that in the RPMI-8226 model, one mouse developed weakness symptoms on day 94, apparently by a non-tumor related cause, and we decided to stop treatment administration in all groups at this time. This mouse died one week later and, although we cannot discard toxicity, it should be noted that this would be very long-term toxicity. To further evaluate the potential toxicity of the PIM-Pd combination, we also carried out experiments with donors’ and patients’ peripheral blood samples treated ex vivo with single drugs, doublets, and triplets (Figures S5 and S6). As can be observed, data indicate that the toxicity of the PIM-Pd combination on lymphocytes and granulocytes is mild to moderate, and not especially higher than that observed with double combinations. On monocytes, the toxicity of PIM-Pd seems to be more pronounced and slightly higher than that observed with double combinations, especially with longer exposition (48 h).

We also evaluated the induction of apoptosis by TUNEL assay in tumor sections from mice treated for two consecutive days with PIM447, Pd or PIM-Pd. Of note, whereas PIM447 or Pd treatments only stabilized tumor growth, the PIM-Pd combination was able to reduce tumor volume by almost 30% (Figure S7). Moreover, quantification of TUNEL positive cells showed that the triple combination induced a significantly (*p* < 0.01) higher percentage of apoptotic cells than PIM447 alone or Pd (Figure 2E).

### 2.3. The PIM-Pd Combination Inhibits Global Protein Synthesis in Myeloma Cells by Targeting the mTORC1 Pathway and Impairing eIF4E Function

**Figure 3:**
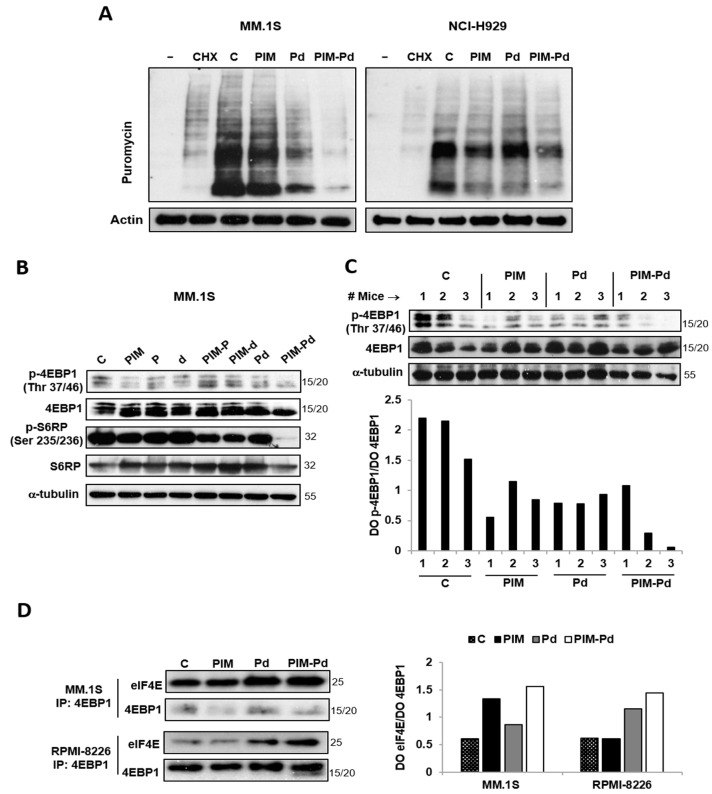
The PIM-Pd combination inhibits global protein synthesis in myeloma cells. (**A**) The SUnSET technique relies on the incorporation of puromycin into nascent proteins and its subsequent detection with an anti-puromycin antibody. MM.1S and NCI-H929 cells were incubated for 24 h in the absence (control; C) or presence of the indicated treatments and then exposed to puromycin (10 μg/mL) for 30 min. Cellular extracts were prepared and analyzed by immunoblotting with an antibody raised against puromycinylated proteins using actin as loading control. The “-” condition indicates a control without puromycin. A condition pre-incubated with cycloheximide (CHX, 50 μM) for 30 min before adding puromycin was also included. (**B**) MM.1S cells were incubated for 48 h in the absence (control; C) or presence of the indicated treatments. The expression of p-4EBP1 (phosphorylated at Thr 37/46), 4EBP1, p-S6RP (phosphorylated at Ser 235/236), and S6RP was evaluated by Western blot. Loading control: α-tubulin. (**C**) Expression of p-4EBP1 (Ser 235/236) and 4EBP1 in protein samples obtained from large MM.1S plasmacytomas of mice treated for two consecutive days with the vehicle (control; C), PIM, Pd, and PIM-Pd as indicated in the Methods section. Loading control: α-tubulin. Bands corresponding to p-4EBP1 and 4EBP1 were quantified by densitometry analysis (using ImageJ software, National Institutes of Health, Bethesda, Maryland, USA) and normalized to α-tubulin, and the ratio p-4EBP1/4EBP1 was calculated. (**D**) Protein extracts were prepared from MM.1S and RPMI-8226 cells untreated (control; C) or treated for 48 h with PIM, Pd or PIM-Pd. The extracts were subjected to immunoprecipitation (IP) with an anti-4EBP1 antibody and analyzed by western blot using an anti-eIF4E antibody (**left panel**). Bands were quantified by densitometry analysis (ImageJ software), and eIF4E bound to 4EBP1 was represented as the ratio eIF4E/4EBP (**right panel**). Doses of PIM/P/d used in (**A**,**B**,**D**) (in nM): 100/200/5 for MM.1S; 100/250/2.5 for RPMI-8226; 200/500/5 for NCI-H929.

We previously demonstrated that PIM447 inhibits mTORC1 [6], which constitutes a master regulator of cell growth through the control of protein translation [14]. Here, we observed that treatment of MM.1S and NCI-H929 cells with PIM447 inhibits global protein biosynthesis, an effect also observed with the double combination Pd and to a greater extent with the triple combination PIM-Pd (Figure 3A). The effect of PIM-Pd by inhibiting protein translation can also be observed on RPMI-8226 cells (Figure S8A). mTORC1 regulates protein translation by phosphorylating the eIF4E-binding protein 1 (4EBP1), which impairs its binding to eIF4E, and also by controlling the phosphorylation of the S6 ribosomal protein (S6RP) [15,16]. Accordingly, treatment of MM.1S cells with PIM-Pd markedly reduced the levels of phospho-4EBP1 (Thr 37/46) and phospho-S6RP (Ser 235/236) as compared to individual treatments and double combinations (Figure 3B). Similarly, the triple combination also reduced the levels of phospho-4EBP1 (Thr 37/46) in NCI-H929 and RPMI-8226 cells; however, it only reduced phospho-S6RP (Ser 235/236) levels in NCI-H929 (Figure S8B). Additionally, levels of phospho-4EBP1 (Thr 37/46) were also lower in MM.1S plasmacytomas of two out of three mice treated with PIM-Pd for two consecutive days as compared to those from mice treated with the vehicle (control, C), PIM447 alone or the Pd doublet (Figure 3C). 

Considering the above results, we evaluated the binding of eIF4E to 4EBP1 after treatment with PIM-Pd by immunoprecipitation assays. Treatment of MM.1S and RPMI-8226 cells with the triple combination increased the binding of eIF4E to 4EBP1 as compared to untreated control cells and cells treated with PIM447 or with Pd (Figure 3D).

### 2.4. Treatment of Myeloma Cells with PIM-Pd Reduces the Expression of the Survival Factors c-Myc and IRF4

**Figure 4:**
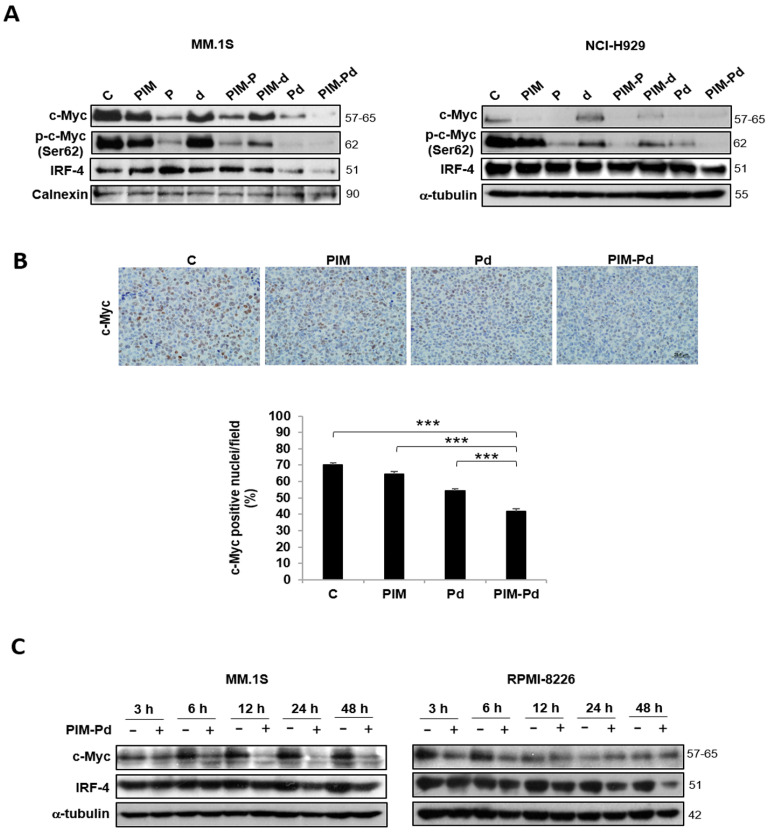
Treatment of myeloma cells with PIM-Pd reduces the expression of the survival factors c-Myc and IRF4. (**A**) Expression of c-Myc, p-c-Myc (phosphorylated at Ser62), and IRF4 analyzed by Western blot in MM.1S and NCI-H929 cells after incubation in the absence (control; C) or presence of the indicated treatments for 48 h. (**B**) Representative images (60× magnification) of immunohistochemical staining of c-Myc in tumor sections isolated from mice treated for two consecutive days with the vehicle (control; C), PIM, Pd or PIM-Pd as specified in the Methods section. Scale bar = 20 µm. Bar chart represents the percentage of c-Myc-positive nuclei per field quantified using the ARIOL automated image analysis system (Leica Biosystems, Wetzlar, Germany). Statistically significant differences among treatment groups and pairwise comparisons were evaluated by Kruskal–Wallis test. Differences between PIM-Pd treatment and all other conditions are indicated: *** *p* < 0.001. (**C**) MM.1S and RPMI-8226 cells were incubated in the absence (−) or presence (+) of the PIM-Pd combination for 3, 6, 12, 24, and 48 h, and the expression of c-Myc and IRF4 was evaluated by Western blot. Loading control: α-tubulin. In **A** and **C** doses of PIM/P/d were (in nM): 100/200/5 for MM.1S; 100/250/2.5 for RPMI-8226; 200/500/5 for NCI-H929.

Both PIM447 in monotherapy and the standard-of-care Pd have been reported to reduce c-Myc levels [6,17]. Consequently, the PIM-Pd combination downregulated the global levels of c-Myc and also those of phospho-c-Myc (Ser62) in MM.1S and NCI-H929 cells after 48 h of treatment (Figure 4A). It should be noted that the ratio phospho-c-Myc/c-Myc decreased with the triple combination in NCI-H929 but not in MM.1S (Figure S9) suggesting that, at least in this last cell line, the decrease of global phospho-c-Myc (Ser62) levels is a consequence of the reduction of global c-Myc levels. IRF4, a MM pro-survival factor [18], was also downregulated with the triple combination (Figure 4A). In line with these in vitro results, MM.1S plasmacytomas displayed a significantly lower percentage of c-Myc-positive nuclei after treatment with PIM-Pd, as compared to PIM and Pd (Figure 4B). In RPMI-8226 cells, however, the triple combination did not modify the expression of either c-Myc or phospho-c-Myc at 48 h, but it reduced the expression of IRF4 (Figure S10A,B). 

Since *MYC* is a direct target of IRF4 in activated B cells and myeloma [18], we next evaluated the expression of both proteins after treatment with PIM-Pd over time. Our results showed that 6 h of treatment with PIM-Pd was sufficient to reduce the expression of c-Myc in MM.1S cells; this reduction was maintained until 48 h later; on the contrary, the downregulation of IRF4 was only detectable after longer exposure to PIM-Pd, specifically at 24 and 48 h of treatment (Figure 4C). Similar results were observed in the RPMI-8226 cell line: a rapid reduction of c-Myc with the triple combination (at 3 h) and a reduction of IRF4 at longer times of treatment (24–48 h). However, in contrast to MM.1S, c-Myc levels were very similar in untreated and PIM-Pd treated RPMI-8226 cells from 12 h onwards (Figure 4C). Since the decrease of c-Myc occurs earlier in time than the reduction of IRF4, we suggest that the decrease of c-Myc protein levels observed after PIM-Pd treatment is not only a consequence of IRF4 downregulation, but also probably a direct effect of the inhibition of cap-dependent translation, of which MYC is one of its principal targets [19].

### 2.5. The PIM-Pd Combination Deregulates Pathways Involved in the Cell Cycle, Gene Expression, Metabolism of RNA and Energy Metabolism

**Figure 5:**
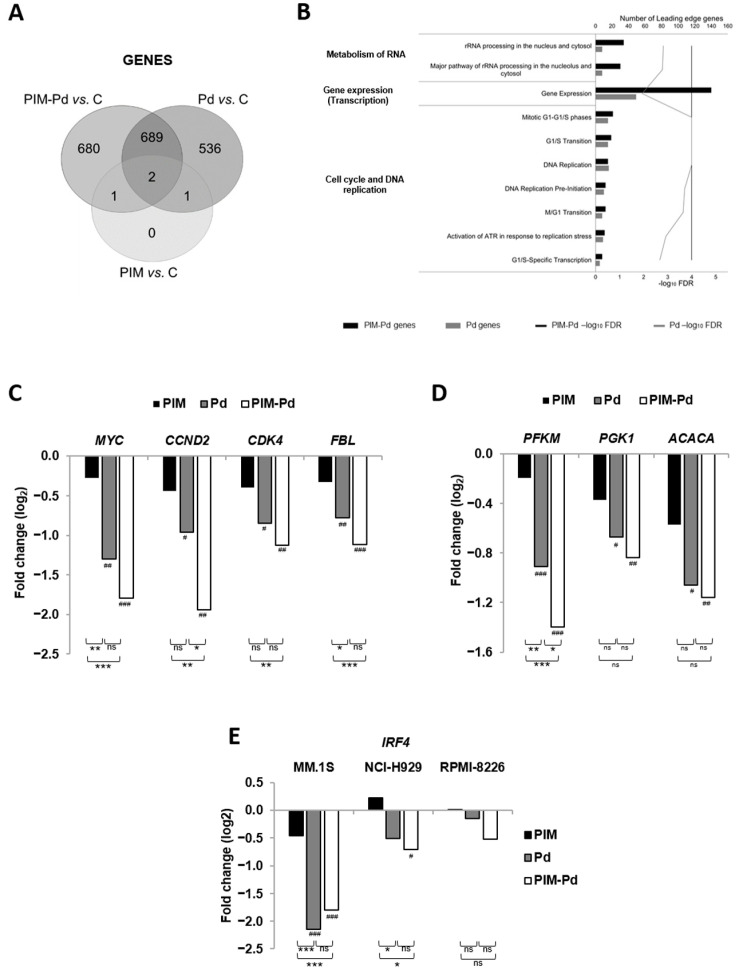
Treatment of myeloma cells with PIM-Pd deregulates pathways involved in the cell cycle, gene expression, metabolism of RNA, and energy metabolism. RNA was isolated from three independent experiments of MM.1S cells untreated (control; C) or sub-lethally treated (% viability > 74%) with: PIM (PIM447 100 nM, for 24 h), Pd (pomalidomide 200 nM + dexamethasone 5 nM, for 15 h) or PIM-Pd (PIM447 100 nM + pomalidomide 200 nM + dexamethasone 5 nM, for 10 h). Subsequently, samples were hybridized to ClariomTM S Assay, human, according to Affymetrix protocols. (**A**) Venn diagram of the significantly deregulated genes after treatment with PIM, Pd, and PIM-Pd vs. control. (**B**) Graph showing in black bars the ten most significantly deregulated pathways after PIM-Pd treatment together with the number of leading edge genes and the significance of each one expressed as –log10 FDR. Data for Pd treatment are represented in grey bars. The TopLevel pathways, according to the Reactome database, are indicated on the left side. (**C**–**E**) mRNA levels of *MYC*, *CCND2* (cyclin D2), *CDK4*, *FBL* (fibrillarin), *PFKM* (phosphofructokinase), *PGK1* (phosphoglycerate kinase), *ACACA* (acetyl-CoA carboxylase), and *IRF4* were assessed by RT-qPCR. The results are shown as the magnitude of change between treated and untreated cells after normalization with 18S rRNA and correspond to the average of three experiments. Statistically significant differences among groups were evaluated by one-way ANOVA followed by Tukey´s HSD post-test. Pairwise differences between treatment groups are indicated as *** *p* < 0.001, ** *p* < 0.01, and * *p* < 0.05. Pairwise differences between each treatment group and control (untreated) condition are indicated as ### *p* < 0.001, ## *p* < 0.01, and # *p* < 0.05. Not significant = ns. Doses of PIM/P/d were (in nM): 100/200/5 for MM.1S; 100/250/2.5 for RPMI-8226; 200/500/5 for NCI-H929.

The transcriptomic profile of MM.1S cells sub-lethally treated with PIM447, Pd, and PIM-Pd (Figure S11) was next analyzed. Compared to untreated control cells and using as a cut off a fold change ≥2 and a *q*-value < 5%, PIM447 treatment only deregulated four genes, Pd treatment deregulated 1228 genes, and the triple combination deregulated 1372 genes, 680 of them being exclusive to this treatment (Tables S4–S6 and Figure 5A). Moreover, the number of pathways significantly (*p* < 0.05) deregulated with PIM-Pd in a gene set enrichment analysis (GSEA) was clearly higher than that deregulated with Pd (67 vs. 39).

The ten most significantly deregulated pathways with PIM-Pd (all under-expressed) were included in the following Top-Level pathways according to Reactome: “Metabolism of RNA”, ”Gene Expression”, ”Cell cycle”, and ”DNA Replication”. Although these ten pathways are also deregulated with Pd, it should be highlighted the higher significance level as well as the higher number of leading edge genes after treatment with the triplet as compared to the doublet (Figure 5B). Importantly, one of the genes downregulated within these pathways was *MYC*. It was subsequently confirmed by RT-qPCR that PIM-Pd downregulated *MYC* to a greater extent than PIM alone and Pd in MM.1S and NCI-H929 cells (Figure 5C and Figure S12). In RPMI-8226, however, PIM-Pd did not decrease *MYC* but prevented the increase observed with PIM alone and Pd (Figure S12). The downregulation of other interesting genes in MM.1S cells after PIM-Pd treatment was also confirmed by RT-qPCR: *CCND2* and *CDK4* (involved in cell cycle), and *FBL* (implicated in gene expression and RNA metabolism) (Figure 5C). Similar results for *CDK4* and *CCND2* were observed in NCI-H929 cells, while PIM-Pd barely modified these genes in RPMI-8226 cells (Figure S12). 

Interestingly, 32 out of 67 pathways were exclusively deregulated with PIM-Pd (31 downregulated and one upregulated). Among them, the pathway “Metabolism”, contained the highest number of leading edge genes (Figure S13) whose functions are related to glycolysis (e.g., the rate-liming enzyme *PFKM* and the enzyme *PGK1*) and fatty acid biosynthesis (e.g., the rate-liming enzyme *ACACA*), among others. The downregulation of these metabolic genes after PIM-Pd treatment was also confirmed by RT-qPCR in MM.1S cells (Figure 5D). *PFKM* showed a similar tendency to be downregulated in NCI-H929 and RPMI-8226 cell lines after PIM-Pd treatment (Figure S14).

Finally, it is also interesting that microarray analysis showed *IRF4* among the genes downregulated in MM.1S cells after PIM-Pd treatment. This result was also evaluated by RT-qPCR, confirming that treatment with PIM-Pd significantly downregulates *IRF4* at the transcriptional level in MM.1S and NCI-H929 cell lines, and a similar tendency was observed in RPMI-8226 cells (Figure 5E).

### 2.6. Treatment of Myeloma Cells with PIM-Pd Induces Cell Cycle Arrest by Modifying the Levels of G0/G1-Transition Regulators and Reduces Glucose Uptake

**Figure 6:**
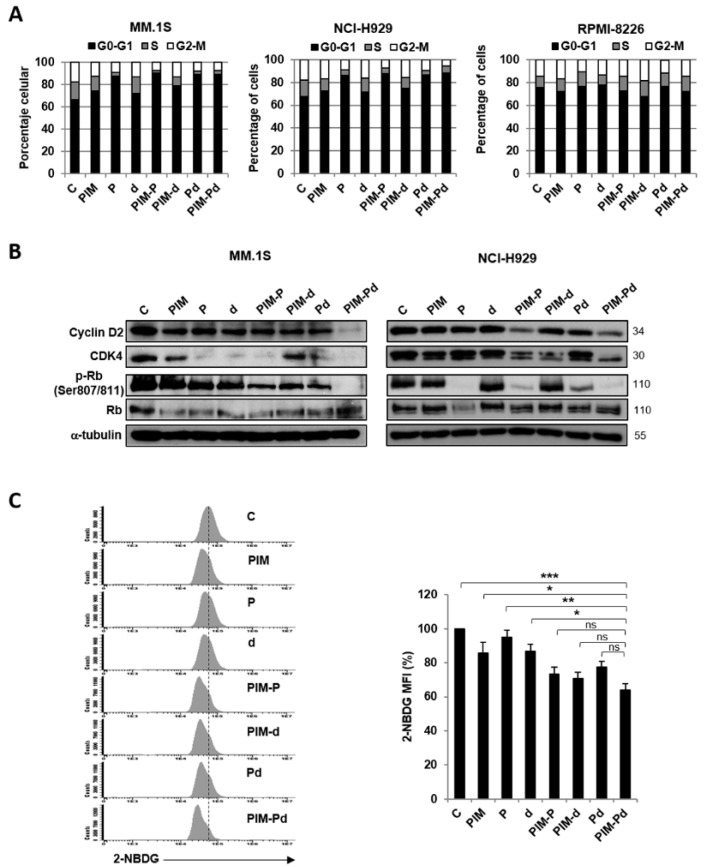
Treatment of myeloma cells with the triple combination PIM-Pd induces cell cycle arrest and reduces glucose uptake. (**A**) Percentage of different phases of the cell cycle in MM.1S, NCI-H929, and RPMI-8226 cell lines after incubation in the absence (control; C) or presence of PIM447 (PIM), pomalidomide (P), dexamethasone (d) or the corresponding double and triple combinations for 72 h. Data represent the means of three independent experiments. (**B**) Expression of cyclin D2, CDK4, Rb, and p-Rb (phosphorylated at Ser807/811) in MM.1S and NCI-H929 cell lines after incubation in the absence (control; C) or presence of the indicated treatments for 48 h. (**C**) Left panel: representative flow cytometry histograms showing the 2-NBDG uptake by MM.1S cells untreated (control; C) or treated with the indicated treatments for 24 h. Right panel: 2-NBDG MFI expressed as a percentage with respect to the control condition. Data represent the mean ± SEM of three independent experiments. Statistically significant differences among different conditions were evaluated by one-way ANOVA followed by Tukey´s HSD post-hoc test. Differences between PIM-Pd treatment and all other conditions are indicated: * *p* < 0.05; ** *p* < 0.01; *** *p*< 0.001; ns = not significant. In A, B, and C, doses of PIM/P/d were (in nM): 100/200/5 for MM.1S; 100/250/2.5 for RPMI-8226; 200/500/5 for NCI-H929.

In line with transcriptomic data, we observed that PIM-Pd treatment induced in MM.1S and NCI-H929 cell lines a cell cycle blockade with an increase in the percentage of cells in G0–G1 phases and a decrease of proliferative phases (S and G2–M); however, no clear effects on cell cycle were observed in the RPMI-8226 cell line after PIM-Pd treatment (Figure 6A). 

Cyclin-dependent kinases 4 and 6 (CDK4 and CDK6) associate with D cyclins to induce the inactivation of the retinoblastoma (Rb) protein and ultimately promote cell-cycle entry and progression through G1 [20]. In accordance with the transcriptomic downregulation of CDK4 and CCND2 (cyclin D2) after PIM-Pd treatment, we also found that the triple combination reduced CDK4 and cyclin D2 at the protein level in MM.1S and NCI-H929 cell lines (Figure 6B). Moreover, PIM-Pd treatment noticeably reduced the phosphorylation of the Rb protein as compared to individual treatments and double combinations that would explain the observed inhibition of cell cycle progression (Figure 6B). 

It is known that tumor cells reprogram metabolic pathways, including glycolysis, to meet their needs for proliferation [21]. Considering the deregulation of metabolic pathways by PIM-Pd according to transcriptomic analysis, we evaluated the effect of the combination on glucose uptake. Thus, treatment of the MM.1S cell line with PIM-Pd significantly reduced the uptake of the 2-NBDG glucose analog with respect to individual treatments and also showed a tendency to reduce it with respect to double combinations (Figure 6C).

## 3. Discussion

PIM kinases, especially PIM2, have been postulated as therapeutic targets in MM due to their overexpression in this disease [2,6,22]. Amongst the different PIM inhibitors developed, PIM447 represents a particularly attractive one due to its activity as a Pan-PIM inhibitor and also due to the fact that it is the first of its class to have reached clinical development as a single agent in MM with promising preliminary efficacy results [9].

In the present work, we demonstrate the potent effect of the triple combination PIM-Pd in delaying tumor growth and prolonging survival in human plasmacytoma murine models. Considering these promising data, the future evaluation of this combination in PDX models of myeloma would be highly interesting due to the predictive value that these models have on patients’ response. Based on our data, we infer that the potent anti-myeloma effect observed with the PIM-Pd combination is, at least partially, induced via the inhibition of global protein synthesis. We consider this effect to be of critical relevance since protein synthesis is frequently deregulated in cancer cells to support aberrant cell growth and proliferation [23]. Moreover, it has been previously shown that PIM447-sensitive DLBCL cells use PIM kinases to maintain activation of translation which is inhibited upon PIM447 treatment [24]. Eukaryotic protein synthesis has several mechanisms to initiate translation, with cap-dependent translation being the pathway used by the majority of mRNAs [25]. Cap-dependent translation is primarily regulated by the heterotrimeric protein complex eIF4F which is composed of the scaffolding protein eIF4G1, the RNA helicase eIF4A1, and the eIF4E factor that binds to the 5’ cap of mRNAs for the recruitment of ribosomes [16]. It is known that mTORC1 controls eIF4F assembly by liberating eIF4E from its respective inhibitory binding protein, 4E-BP1 [14]. Thus, part of the inhibitory effect of PIM-Pd on protein synthesis could be explained by the observed inhibition of the mTORC1 pathway, both in vitro and in vivo, which results in an increased binding between eIF4E and 4E-BP1 due to the decrease of phospho-4E-BP1 levels. It should be noted that mTORC1 controls global protein synthesis but, at the same time, previous studies have demonstrated that fluctuations of eIF4E levels mainly affect the translation of mRNAs harboring long, highly structured 5′-UTRs, which encode proteins that control cell proliferation and viability [19,26,27]. In line with these observations, our results show that treatment with PIM-Pd decreases the levels of c-Myc and cyclin D2, two well-known regulators of cell cycle [28,29], whose levels have been previously reported to decrease in myeloma cells when cap-dependent translation is selectively inhibited [30]. Moreover, the transcriptional downregulation of *MYC* and *CCND2* (cyclin D2) would also contribute to reduce both molecules at the protein level. However, we cannot conclude, at least not in all cell lines evaluated, that treatment with PIM-Pd decreases the ratio of c-Myc phosphorylated at Ser62/c-Myc (which would lead to a less stabilized c-Myc). Rather, the decrease of global phospho-c-Myc (Ser62) levels seems to be more a consequence of the reduction of global c-Myc levels. The downregulation of c-Myc and cyclin D2 together with the reduction of CDK4 and phospho-Rb levels is in agreement with the G0/G1 cell cycle arrest induced by the triple combination. In fact, pathways related to the cell cycle are among the top ten deregulated in the transcriptomic analysis after treatment with PIM-Pd. It remains to be elucidated why in the RPMI-8226 cell line the downregulation of c-Myc after treatment with PIM-Pd is not maintained stable over time, but it is likely that this event contributes to the absence of cell cycle changes in this cell line. Nonetheless, we assume that the inhibition of protein synthesis by decreasing phospho-4EBP1 together with the downregulation of the survival factor IRF4 (these effects also being observed in MM.1S and NCI-H929) contributes to reduce the viability of RPMI-8226 cells after PIM-Pd treatment.

Apart from controlling the cell cycle, c-Myc also regulates the transcription of metabolic genes [31,32,33]. In line with this, the decrease of c-Myc after PIM-Pd treatment could mediate the transcriptional downregulation of the glycolytic enzymes PFKM (phosphofructokinase) and PGK1 (phosphoglycerate kinase) and the enzyme involved in fatty acid biosynthesis ACACA (acetyl-CoA carboxylase), since all of them have been previously described as direct targets of c-Myc [31,32,33]. Moreover, our results indicate that the triple combination reduces glucose uptake by myeloma cells.

Another important function of c-Myc is the control of protein synthesis through the direct regulation of the transcription of ribosomal RNA (rRNA) genes and genes required for rRNA processing and assembly [34]. In fact, pathways involved in rRNA processing are also among the top ten deregulated after treatment with PIM-Pd. Interestingly, the triple combination reduces the transcriptional expression of *FBL* (fibrillarin), an enzyme involved in the first step of pre-ribosomal rRNA processing [35] which has been previously reported to be a direct target of c-Myc [36]. These effects probably contribute, as a feedback loop mechanism, to further potentiate the reduction of protein translation induced by the inhibition of mTORC1. In fact, it has been previously reported that c-Myc and mTOR converge on a common node in protein synthesis control that confers synthetic lethality in Myc-driven cancers, including multiple myeloma [37]. Moreover, other therapies also targeting c-Myc by the inhibition of protein translation have been found to be effective in MM in preclinical studies [38]. Considering that in MM: (i) the occurrence of mutations in genes involved in protein translation is frequent [39], (ii) protein biosynthesis is one of the most significantly upregulated biological process in MM vs. normal plasma cells [40], and (iii) c-Myc has a critical role in the biology of MM [41,42,43], we propose the synergistic inhibition of the oncogenic translation program, by the convergent down-modulation of mTORC1 and c-Myc, as one of the main causes of the anti-myeloma effect of the PIM-Pd combination.

## 4. Materials and Methods 

### 4.1. Drugs

PIM447 (PIM) was provided by Novartis Pharmaceuticals, Inc. (Basel, Switzerland). Pomalidomide (P) was purchased from Selleckchem (Houston, TX, USA) and dexamethasone (d) from Sigma–Aldrich (St Louis, MO, USA).

### 4.2. Cell Lines and Cultures 

The origin of the myeloma cell lines MM.1S, NCI-H929, RPMI-8226, OPM-2, and MM.1S-luc (luciferase-expressing) was previously described [6]. JJN3 was obtained from DSMZ (Braunschweig, Germany). Myeloma cell lines were cultured as described [6] and their identity confirmed by STR analysis with the PowerPlex 16 HS System kit (Promega) and online STR matching analysis (www.dsmz.de/fp/cgi-bin/str.html). The MM.1S-luc co-cultures with either the HS-5 cell line (purchased from ATCC, Manassas, VA, USA) or mesenchymal stromal cells obtained from the bone marrow of MM patients (BM-MSCs) were performed as reported [44]. Primary samples were obtained after approval of the Complejo Asistencial Universitario de Salamanca Review Board (E.O.: 08/91) on 26 May 2008, and after written informed consent of participating subjects, following the Declaration of Helsinki guidelines.

### 4.3. MTT, Cell Cycle, and Apoptosis Assays

MTT, cell-cycle, and apoptosis assays were performed as previously described [6]. In vitro synergism was quantified as described [6] obtaining a combination index (CI) with the following interpretation: CI > 1, antagonistic effect; CI = 1, additive effect; and CI < 1, synergistic effect. The ex vivo analysis of apoptosis induced by treatments on primary myeloma cells obtained from bone marrow samples of patients was performed as previously described [6].

### 4.4. Western Blot and Immunoprecipitation 

Protein lysis and Western blot were performed following standard procedures [6]. The complete list of antibodies is shown in Table S7. For immunoprecipitation assays, equal concentrations of cleared lysates were subjected to immunoprecipitation with an anti-4EBP1 antibody. Immunocomplexes were captured through overnight incubation at 4 °C with protein-A sepharose beads (Sigma-Aldrich, St Louis, MO, USA). The immunoprecipitates were analyzed by immunoblotting.

### 4.5. Determination of Protein Biosynthesis Levels 

The SUnSET technique for measuring protein synthesis was previously reported [45]. Briefly, 1 × 10^6^ cells were seeded and, after 24 h, the corresponding treatment was added and incubated for additional 24 h. Finally, 10 μg/mL puromycin (Invitrogen, Carlsbad, CA, USA) was added for 30 min before cells lysis. A negative control of protein synthesis was included: 50 μM cycloheximide (Sigma-Aldrich) was applied for 30 min before puromycin addition. Protein extraction and immunoblotting were performed as described above. The anti-puromycin antibody was obtained from Merck (Darmstadt, Germany).

### 4.6. Transcriptome Profiling Microarrays 

RNA was isolated, purified, and evaluated for integrity as previously described [46]. Labelling and hybridizations to ClariomTM S Assay human, washing, and scanning were performed following Affymetrix protocols (Santa Clara, CA, USA). Microarray data were normalized by the RMA method [47] implemented in the oligo (v.1.44.0) package in R (v.3.5.0) using a custom BrainArray [48] gene reference (Custom CDF, Ensembl version 22). Unsupervised analysis was performed in SIMFIT statistical software (v.7.4.1) using the Euclidean distance as a distance measure and the group average as the linkage method. Differentially expressed genes were identified using the SAM method via Shiny (v. 1.1.0) in R (https://github.com/MikeJSeo/SAM). Statistically significant gene lists were cross-compared using the DrawVenn online tool (http://bioinformatics.psb.ugent.be/webtools/Venn/). Reactome [49] pathway overrepresentation and enrichment analyses were performed in the Webgestalt suite [50]. All microarray data have been deposited in the Gene Expression Omnibus (http://www.ncbi.nlm.nih.gov/geo/; accession number: GSE138440).

### 4.7. Reverse Transcription Quantitative PCR (RT-qPCR)

The reverse transcription reaction was performed using the High-Capacity cDNA Reverse Transcription Kit (Thermo Fisher Scientific, Waltham, MA, USA). TaqMan Gene Expression Assays (Thermo Fisher Scientific) were performed according to manufacturer’s instructions and are specified in Table S8. Normalized gene expression was calculated as 2^−ΔCt^, being ΔCt = Ct (gene) − Ct (RNA18S5).

### 4.8. 2-NBDG Assay

To monitor glucose uptake we used 2-NBDG (ThermoFisher Scientific). MM.1S cells were incubated for 24 h in the absence or presence of the corresponding treatments. Then, the culture medium was removed and replaced with glucose-free medium (reference R1383 from Sigma–Aldrich) plus 2-NBDG (146 µM) for 30 min. Finally, cells were washed twice with cold PBS, incubated with 7AAD for 5 min, and analyzed by flow cytometry. 2-NBDG mean fluorescence intensity (MFI) was determined over the viable population.

### 4.9. Analysis of Drug Toxicity in Hematopoietic Populations

For the evaluation of drug toxicity, peripheral blood samples from healthy donors (*n* = 3) and MM patients (*n* = 3) were lysed with ammonium chloride to remove red blood cells, and white cells were culture with PIM447 (400 nM), pomalidomide (1000 nM), dexamethasone (10 nM) or the corresponding double and triple combinations for 24 and 48 h. After the incubation time, apoptosis induction on lymphocytes, granulocytes, and monocytes was evaluated by flow cytometry after staining with annexin V-FITC and anti-CD45-PercepCy5.5. Primary samples were obtained after approval of the Complejo Asistencial Universitario de Salamanca Review Board (ethical code: E.O.: 08/91) on May 26, 2008, and after written informed consent of participating subjects, following the Declaration of Helsinki guidelines.

### 4.10. Human Plasmacytoma Murine Model 

The human plasmacytoma model in CB17-SCID mice has been previously described [46]. To evaluate treatment efficacy, mice were randomized (4–5 mice/group) to receive the vehicle, PIM447 (50 mg/kg, 5 times/week by oral gavage), pomalidomide (6 mg/kg, 5 times/week, intraperitoneally-IP), dexamethasone (1 mg/kg, Monday–Tuesday, IP), or the corresponding double and triple combinations. 

In an independent experiment to explore the mechanism of action, mice were randomized (4 mice/group) when tumors reached an average volume of 1700 mm^3^ to be treated for two consecutive days with the vehicle, PIM447, pomalidomide + dexamethasone or PIM447 + pomalidomide + dexamethasone (same doses as above). Mice were sacrificed on the third day, and tumor protein and tissue samples were isolated. Terminal deoxynucleotidyl transferase (TdT) dUTP Nick-End Labeling (TUNEL) assay was performed as previously described [51]. Immunohistochemistry of c-Myc was performed using an anti-c-Myc antibody (Abcam, Cambridge, UK). The percentage of c-Myc-positive nuclei was quantified using the ARIOL automated image analysis system (Leica Biosystems, Wetzlar, Germany) on the entire tumor sample excluding necrotic areas. 

All animal experiments were conducted according to European Guidelines (Directive 2010/63/UE) and Spanish laws (RD53/2013) for the use of laboratory animals, and after being granted permission for animal experimentation from the University of Salamanca Animal Ethical Committee and Agriculture and Livestock Council of Junta de Castilla y León, (Registry Number 0000061; Registered User Center: ES372740000046).

### 4.11. Statistical Analyses 

Statistical analyses were performed using SPSS-v23.0 software (IBM Corp., Armonk, NY, USA) as indicated for each experiment. Specifically, differences in tumor growth among groups were analyzed using the log10 relative tumor volume (RTV) [52] as indicated.

## 5. Conclusions

In conclusion, the results presented in this work show the potent anti-myeloma activity of the combination of PIM447, the first and currently only pan-PIM kinase inhibitor to have reached clinical development in MM, with the standard-of-care Pd, and provide some clues to the potential mechanisms involved in this effect. These preclinical data support the clinical development of the triple combination PIM-Pd for the treatment of patients with MM.

## Supplementary Materials

The following are available online at https://www.mdpi.com/2072-6694/12/10/2743/s1. Figure S1: Time kinetics study of the synergy of the PIM-Pd combination in different MM cell lines, Figure S2: Ex vivo effect of the PIM-Pd combination on myeloma cells from patients, Figure S3: The triple combination PIM-Pd does not modify the viability of BM-MSCs, Figure S4: Effect of PIM-Pd administration on body weight in xenograft CB17-SCID mouse models of myeloma, Figure S5: Evaluation of the toxicity of the PIM-Pd combination in hematopoietic populations in healthy donors’ blood samples, Figure S6: Evaluation of the toxicity of the PIM-Pd combination in hematopoietic populations in MM patients’ blood samples, Figure S7: The triple combination PIM-Pd largely reduced the volume of large plasmacytomas after two doses of treatment, Figure S8: Effect of the PIM-Pd combination on global protein synthesis and the mTORC1 pathway in myeloma cells, Figure S9: Ratio p-c-Myc/c-Myc after treatment with PIM447 (PIM), pomalidomide (P), dexamethasone (d) or the corresponding drug combinations, Figure S10: Effect of the PIM-Pd combination on the expression of c-Myc, phospho-c-Myc, and IRF4 in RPMI-8226 cells, Figure S11: Percentage cell viability in samples used to hybridize gene expression microarrays for transcriptomic profiling, Figure S12: Effect of the PIM-Pd combination on the expression of cell cycle-related genes, Figure S13: Pathways exclusively deregulated in MM.1S cells after treatment with PIM-Pd, Figure S14: Effect of the triple combination PIM-Pd on the expression of *PFKM* in NCI-H929 and RPMI-8226 cell lines, Table S1: Combination indices (CI) corresponding to the indicated double or triple combinations of PIM447 (PIM), pomalidomide (P), and dexamethasone (d) in NCI-H929, OPM-2, and JJN3 cell lines. CI values were calculated with Calcusyn software based on data from MTT assay after 72 h of treatment, Table S2: Evaluation of toxicity symptoms in the MM1S plasmacytoma model, Table S3: Evaluation of toxicity symptoms in the RPMI-8226 plasmacytoma model, Table S4: Genes significantly deregulated in MM.1S cells with the PIM-Pd combination compared to untreated control cells, Table S5: Genes significantly deregulated in MM.1S cells with the Pd combination compared to untreated control cells, Table S6: Genes significantly deregulated in MM.1S cells with PIM447 treatment compared to untreated control cells, Table S7: List of antibodies used for immunoblotting, Table S8: TaqMan Gene Expression Assays from Thermo Fisher Scientific.
