# Supplementary material for: Protein Translation Inhibition is Involved in the Activity of the Pan-PIM Kinase Inhibitor PIM447 in Combination with Pomalidomide-Dexamethasone in Multiple Myeloma
Source: Cancers (Basel). 2020 Sep 24;12(10):2743. doi: 10.3390/cancers12102743 (PMC7598606; doi:10.3390/cancers12102743)
Supplement: Supplementary file 1 [file cancers-12-02743-s001.zip › cancers-932840-supplementary-figures-final.docx]

Article

Protein Translation Inhibition is Involved in the Activity of the Pan-PIM Kinase Inhibitor PIM447 in Combination with Pomalidomide-Dexamethasone in Multiple Myeloma

Teresa Paíno, Lorena González-Méndez, Laura San-Segundo, Luis A. Corchete, Susana Hernández-García, Andrea Díaz-Tejedor, Esperanza M. Algarín, Pedro Mogollón, Montserrat Martín-Sánchez, Norma C. Gutiérrez, María-Victoria Mateos, Mercedes Garayoa and Enrique M. Ocio


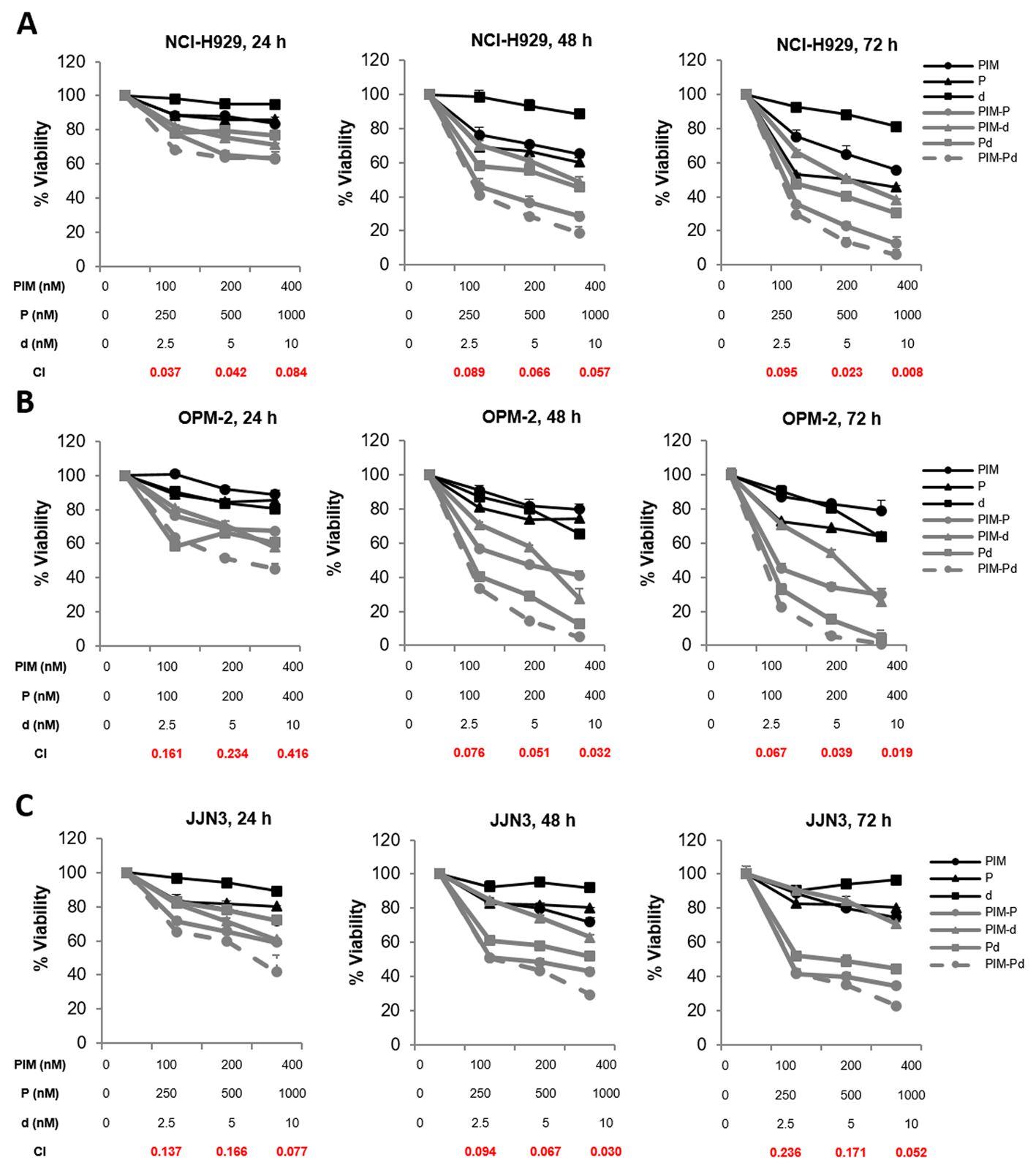


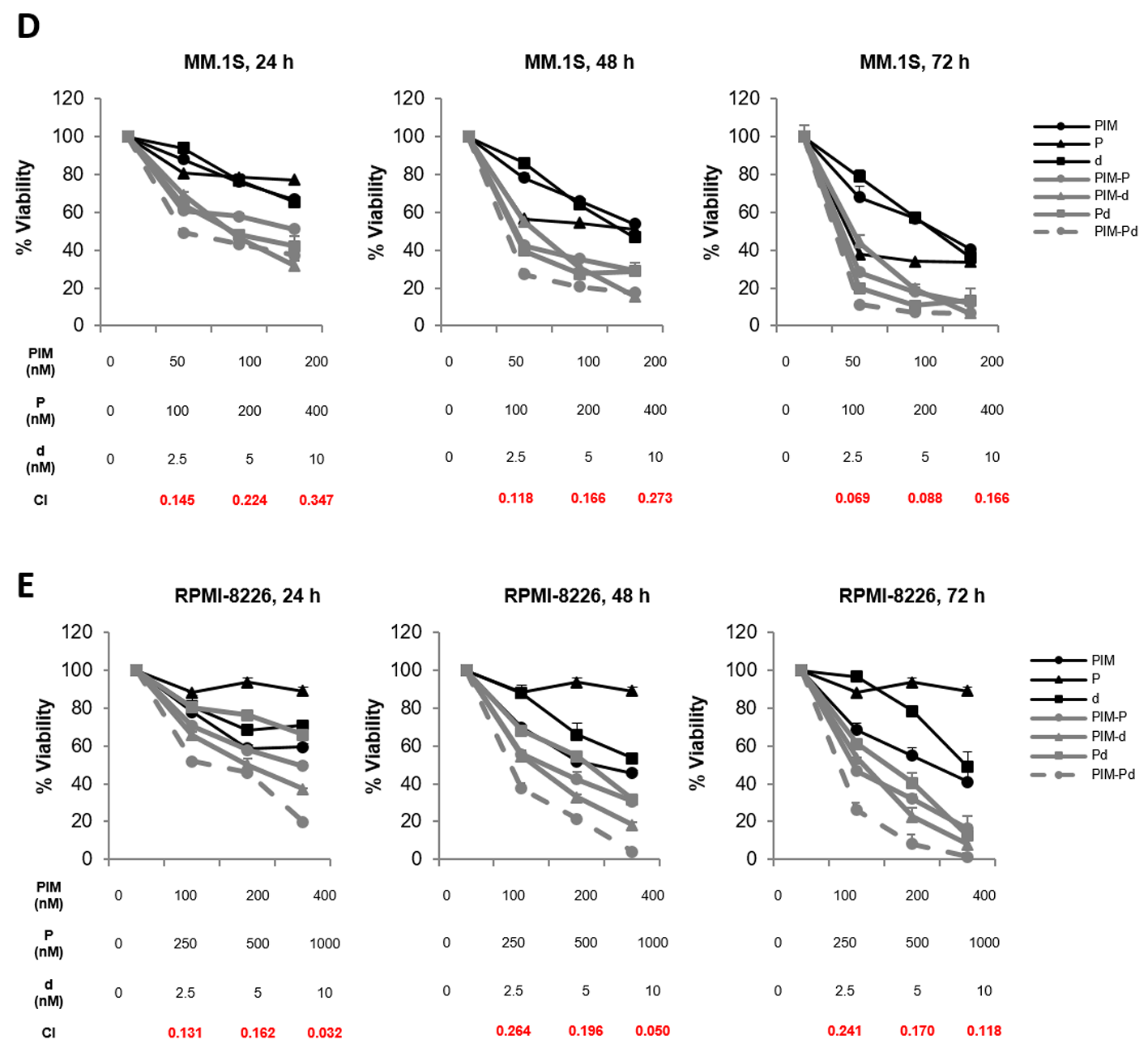


**Figure S1.** Time kinetics study of the synergy of the PIM-Pd combination in different MM cell lines. (**A**) NCI-H929, (**B**) OPM-2, (**C**) JJN3, (**D**) MM.1S and (**E**) RPMI-8226 cell lines were treated for 24, 48 and 72 h with the indicated doses of PIM447 (PIM), pomalidomide (P) or dexamethasone (d) alone or in double and triple combinations, and the percentage of cell viability was calculated based on MTT assay (control,100%). Data represent mean ± SD. CI = combination index corresponding to the PIM-Pd combination.


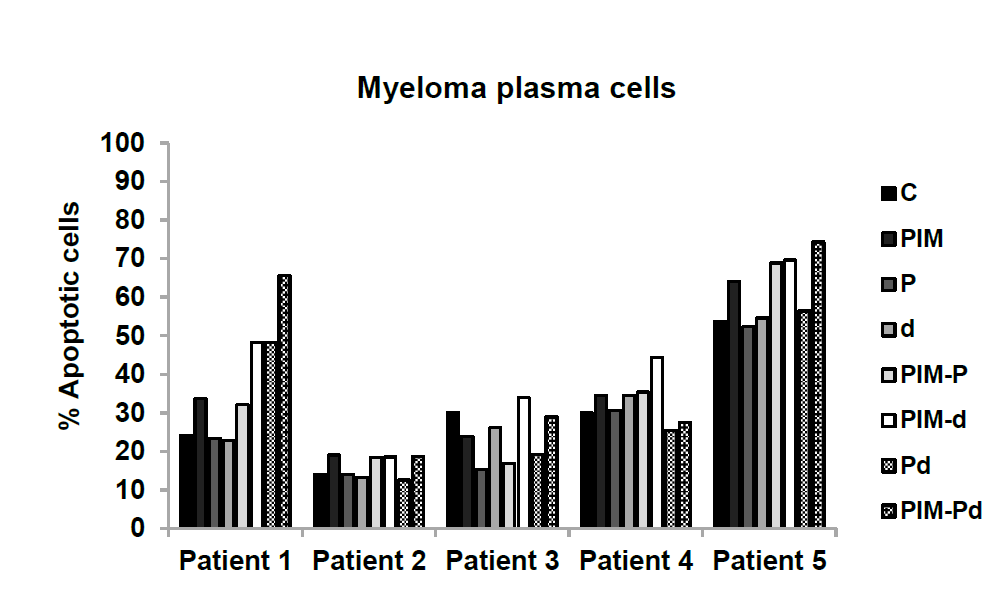


**Figure S2.** Ex vivo effect of the PIM-Pd combination on myeloma cells from patients. Freshly isolated BM cells obtained from 5 patients with multiple myeloma were treated *ex vivo* with PIM447 (PIM), pomalidomide (P) or dexamethasone (d) alone or the corresponding double and triple combinations for 48 h. After the incubation period, cells were stained with the combination of annexin V–FITC and three monoclonal antibodies (CD56-PE, CD45-PerCP-Cy5.5 and CD38-APC) which allowed the evaluation of apoptosis (annexinV^+^) in myeloma cells (CD38^+bright^, CD45^−/low^, SSC^low/intermediate^,CD56^−/+^). Doses: PIM447 (1 μM), pomalidomide (10 μM) and dexamethasone (10 nM).


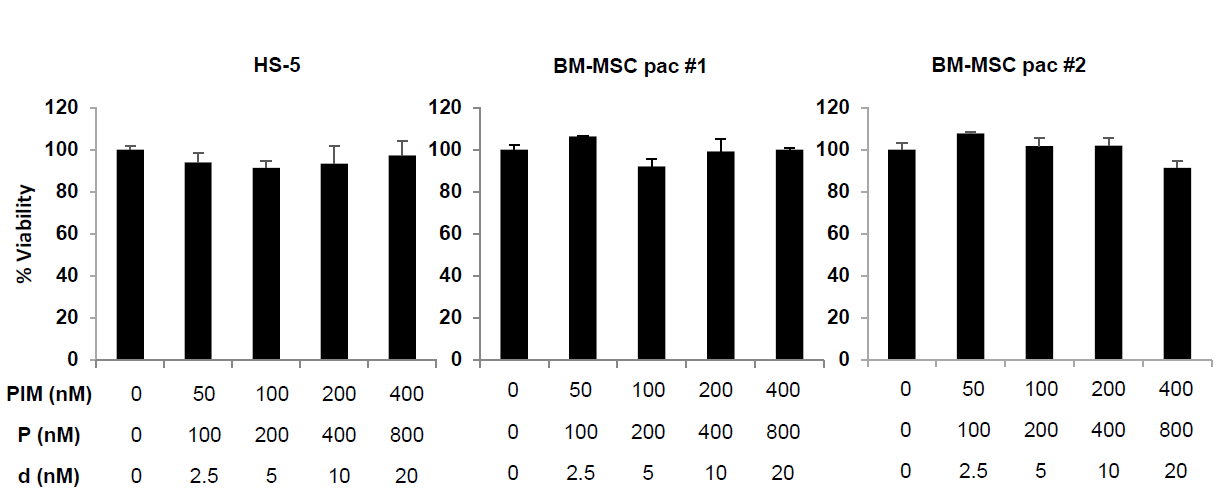


**Figure S3.** The triple combination PIM-Pd does not modify the viability of BM-MSCs. HS-5 cells or BM-MSCs from two patients with MM (pac #1 and pac #2) were treated for 72 h with the triple combination PIM-Pd at the indicated doses, and the percentage of cell viability was calculated based on MTT assay (control,100%).


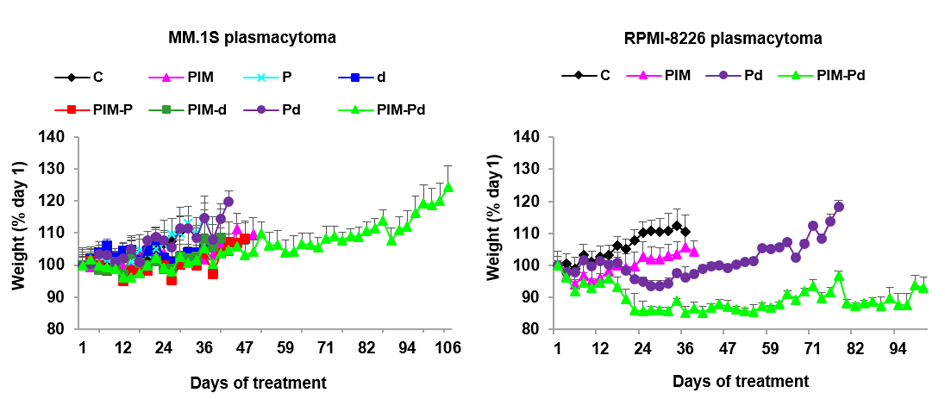


**Figure S4.** Effect of PIM-Pd administration on body weight in xenograft CB17-SCID mouse models of myeloma. The body weight of mice was monitored in the MM.1S (**left**) and RPMI-8226 (**right**) plasmacytoma models throughout the treatment period, and the percentage of weight at each day of treatment was calculated considering day 1 as 100%. Data are summarized as the mean ± SEM.


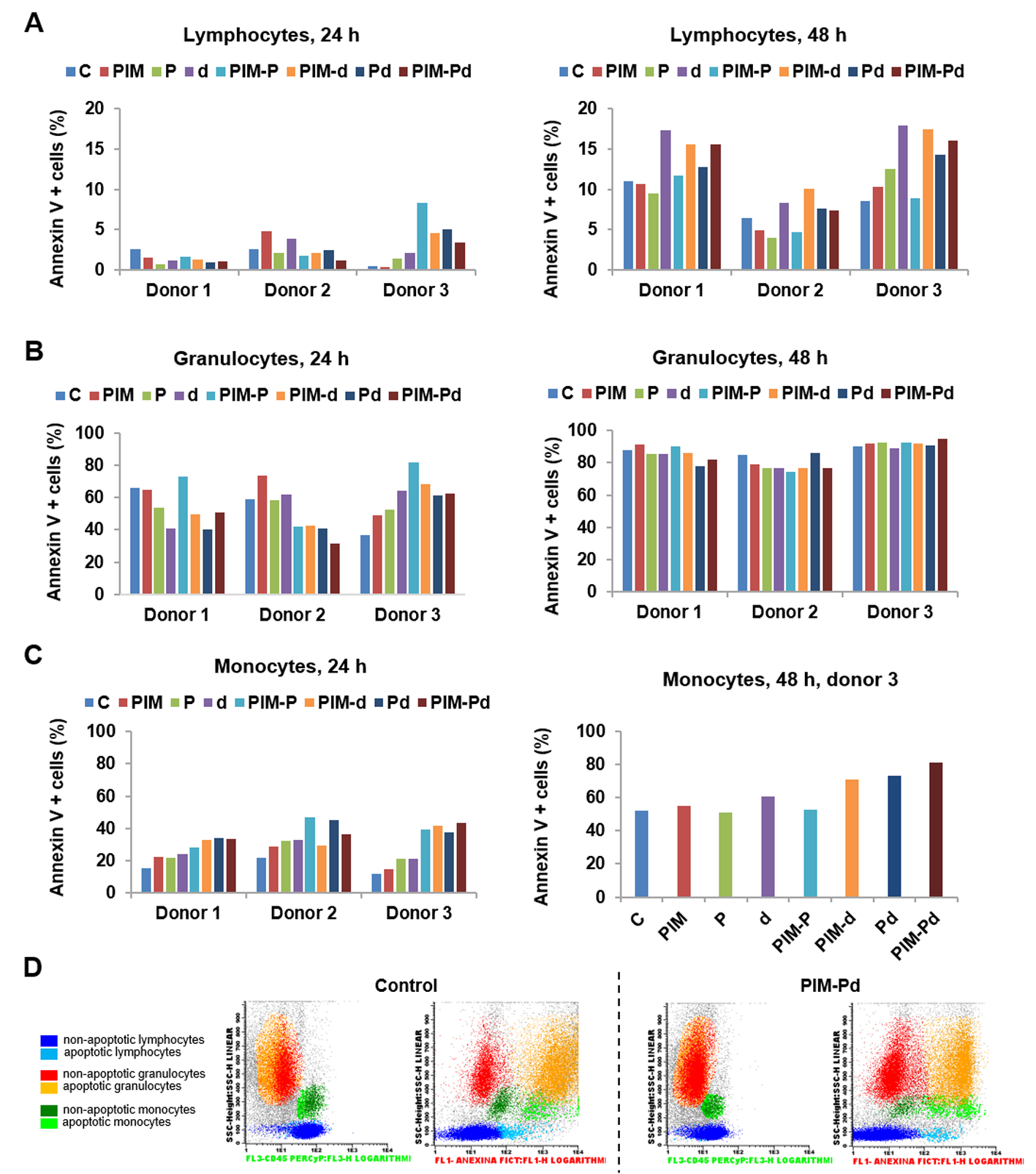


**Figure S5.** Evaluation of the toxicity of the PIM-Pd combination in hematopoietic populations in healthy donors’ blood samples. Peripheral blood samples from three healthy donors were lysed to remove red blood cells and subsequently cultured in absence (control; C) or presence of the indicated treatments. After 24 and 48 h, apoptosis was analyzed by flow cytometry in (**A**) lymphocytes, (**B**) granulocytes and (**C**) monocytes. (**D**) Illustrative dot-plots corresponding to donor1 (24 h) showing the gating strategy. Doses of PIM447 (PIM), pomalidomide (P) and dexamethasone (d) were 400 nM, 1 μM and 10 nM, respectively.


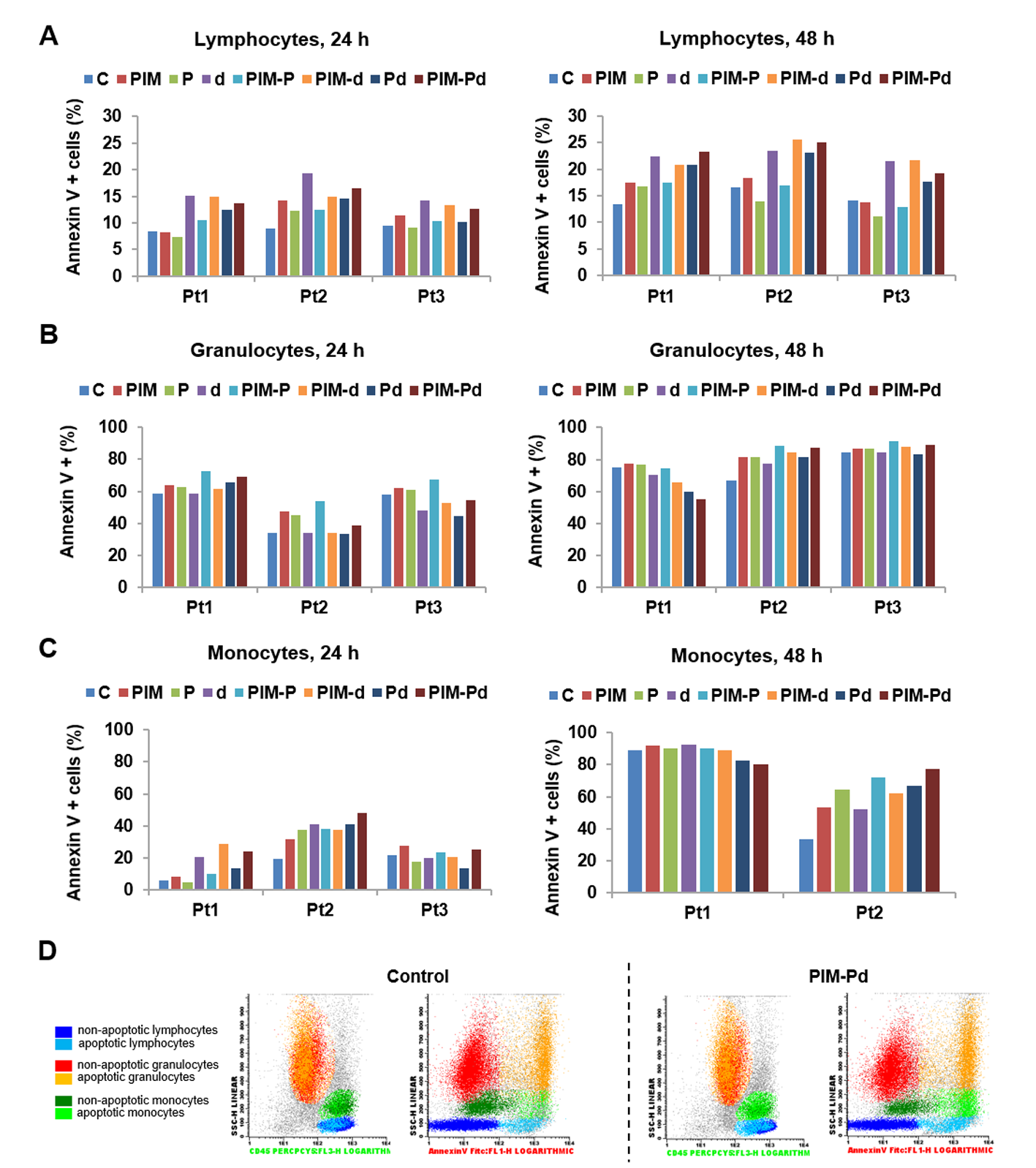


**Figure S6.** Evaluation of the toxicity of the PIM-Pd combination in hematopoietic populations in MM patients’ blood samples. Peripheral blood samples from three MM patients were lysed to remove red blood cells and subsequently cultured in absence (control; C) or presence of the indicated treatments. After 24 and 48 h, apoptosis was analyzed by flow cytometry in (**A**) lymphocytes, (**B**) granulocytes and (**C**) monocytes. (**D**) Illustrative dot-plots corresponding to patient 2 (Pt2, 24 h) showing the gating strategy. Doses of PIM447 (PIM), pomalidomide (P) and dexamethasone (d) were 400 nM, 1 μM and 10 nM, respectively.


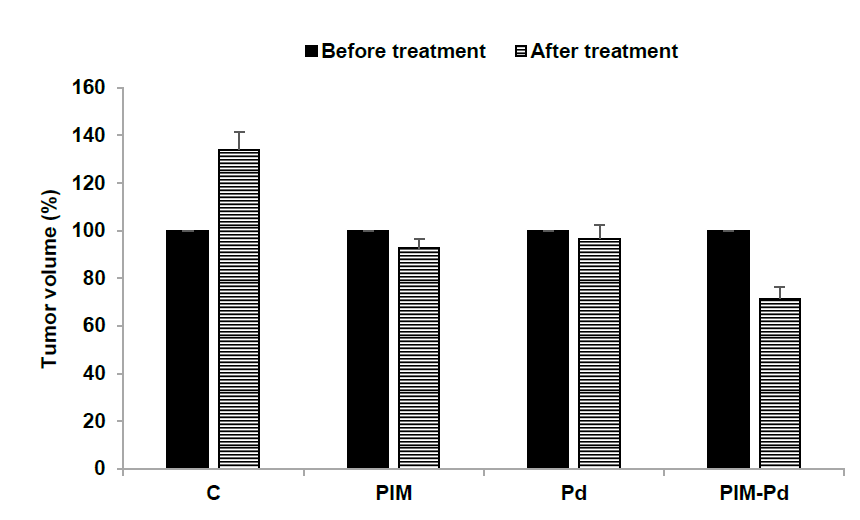


**Figure S7.** The triple combination PIM-Pd largely reduced the volume of large plasmacytomas after two doses of treatment. CB17-SCID mice bearing large MM.1S subcutaneous plasmacytomas (approximately median volume, 1700 mm^3^) were randomly assigned to receive two doses of vehicle (control; C), PIM447 (PIM), pomalidomide + dexamethasone (Pd) or the triple combination PIM-Pd, distributed in two consecutive days (*n* = 3 per group). Graph represents the percentage of tumor volume before and after two doses of treatment, considering the condition “before treatment” as 100%.


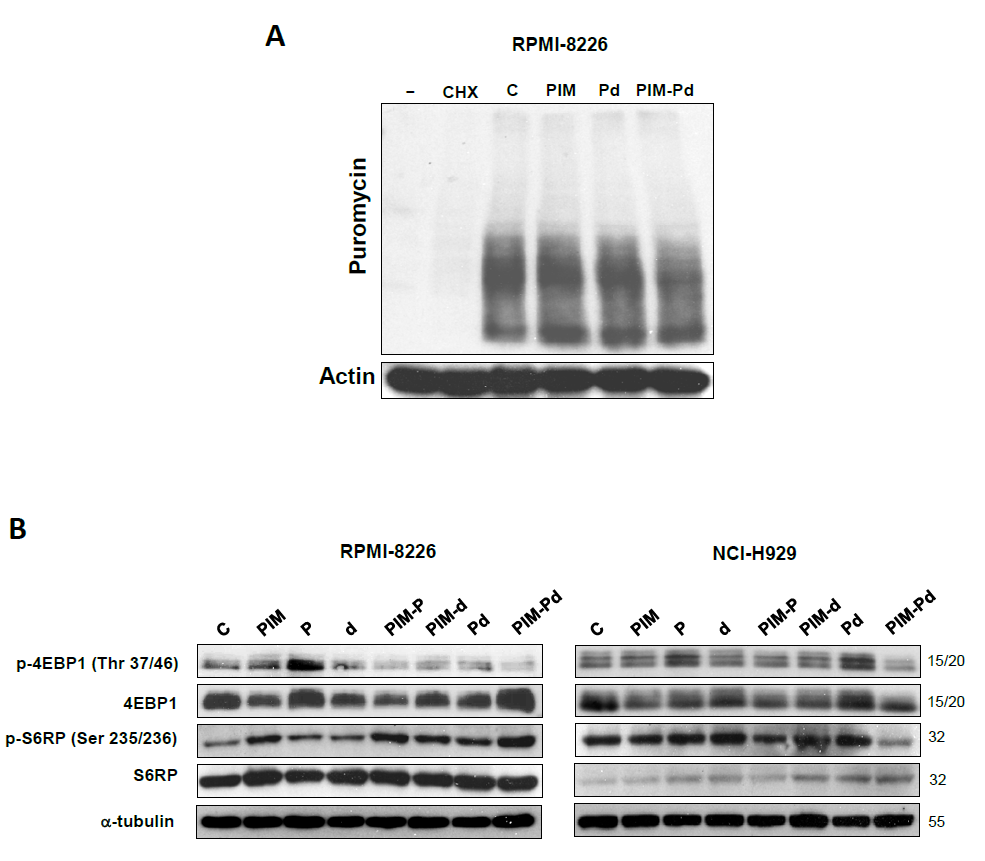


**Figure S8.** Effect of the PIM-Pd combination on global protein synthesis and mTORC1 pathway in myeloma cells. (**A**) RPMI-8226 cells were incubated for 24 h in absence (control; C) or presence of PIM, Pd or PIM-Pd, and then exposed to puromycin (10 μg/mL) for 30 minutes. Cellular extracts were prepared and analyzed by immunoblotting with an antibody against puromycinylated proteins. Loading control: actin. The “-“ condition indicates a control without puromycin. A condition pre-incubated with cycloheximide (CHX, 50 μM) for 30 min before adding puromyc in was also included. (**B**) RPMI-8226 and NCI-H929 cells were incubated for 48 h in absence (C) or presence of the indicated treatments. The expression of p-4EBP1 (phosphorylated at Thr 37/46), 4EBP1, p-S6RP (phosphorylated at Ser 235/236) and S6RP was evaluated by Western blot using α-tubulin as loading control. Doses of PIM/P/d (in nM): 100/250/2.5 for RPMI-8226; 200/500/5 for NCI-H929.


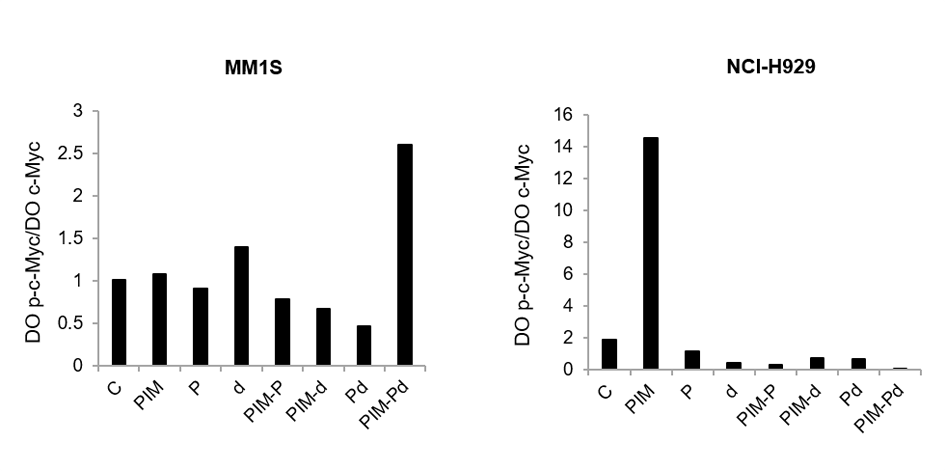


**Figure S9.** Ratio p-c-Myc/c-Myc after treatment with PIM447 (PIM), pomalidomide (P), dexamethasone (d) or the corresponding drug combinations. Bands corresponding to p-c-Myc and c-Myc in Figure 4A were quantified by densitometry analysis (using ImageJ software, National Institutes of Health, Bethesda, Maryland, USA) and normalized to loading control protein, and the ratio p-c-Myc/c-Myc was calculated.


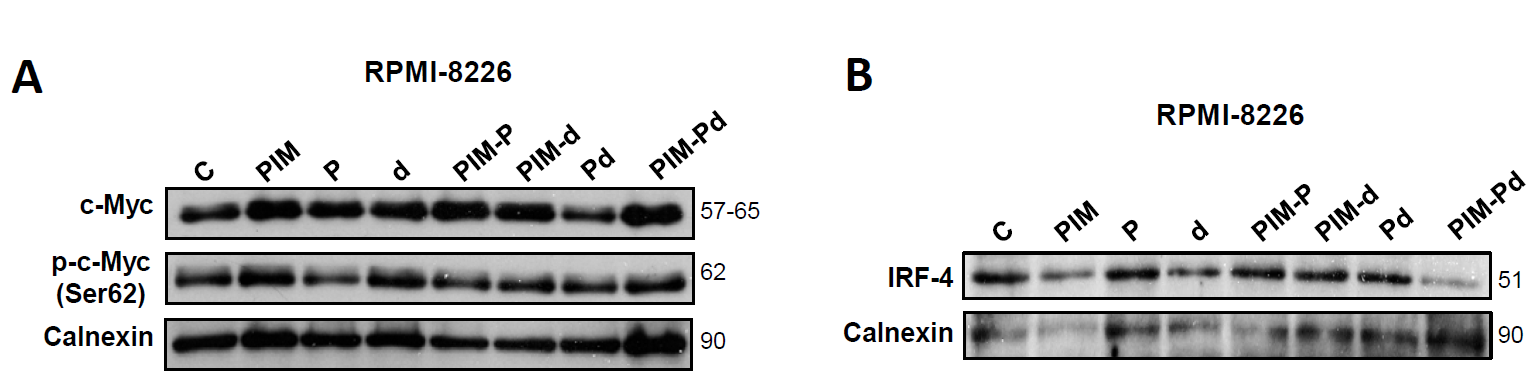


**Figure S10.** Effect of the PIM-Pd combination on the expression of c-Myc, phospho-c-Myc and IRF4 in RPMI-8226 cells. RPMI-8226 cells were incubated in absence(control; C) or presence of PIM447 (PIM; 100 nM), pomalidomide (P; 250 nM), dexamethasone (d; 2.5 nM) or the corresponding double and triple combinations for 48 h, and the expression of c-Myc and p-c-Myc (phosphorylated at Ser62) (**A),** and IRF4 **(B)** was evaluated by Western blot. Calnexin was used as loading control.


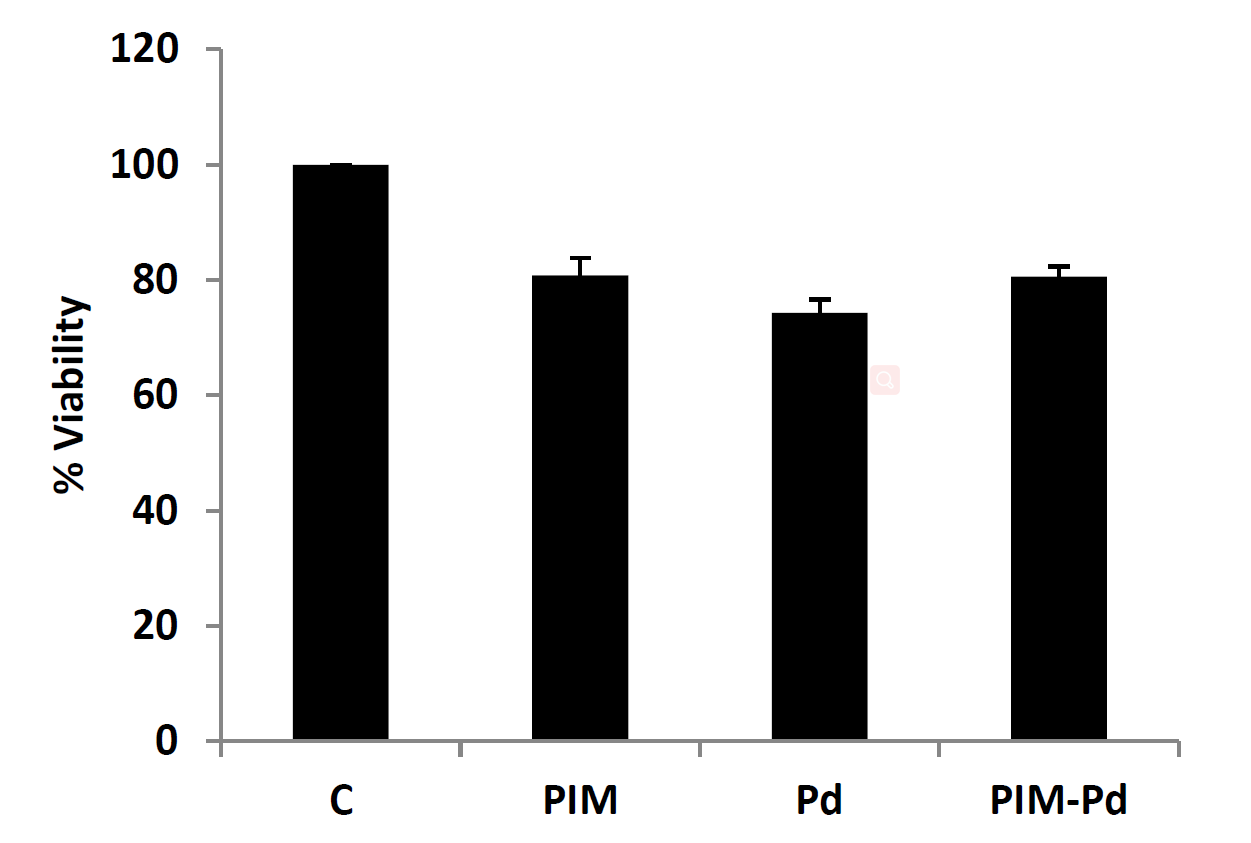


**Figure S11.** Percentage of cell viability in samples used to hybridize gene expression microarrays for transcriptomic profiling. MM.1S cells were sub-lethally treated (%viability >74%) with: PIM (PIM447 100 nM, for 24 h), Pd (pomalidomide 200 nM + dexamethasone 5 nM, for 15 h) or PIM-Pd (PIM447 100 nM + pomalidomide 200 nM + dexamethasone 5 nM, for 10 h). The percentage of cell viability was evaluated by MTT assay with respect to untreated cells (C; control). Data represents mean ± SEM of three independent sets of samples.


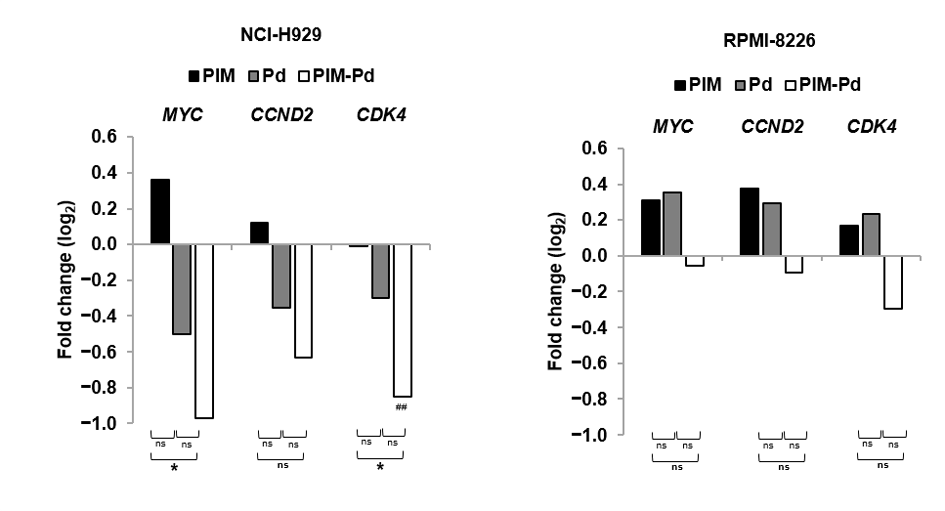


**Figure S12.** Effect of the PIM-Pd combination on the expression of cell cycle related genes. NCI-H929 and RPMI-8226 cells were cultured in absence or presence of PIM447 (PIM), pomalidomide + dexamethasone (Pd) or the triple combination PIM-Pd for 15 h. Subsequently, transcriptomic levels of *MYC, CCND2* and *CDK4* were assessed by RT-qPCR. The results are shown as the magnitude of change between treated and untreated cells after normalization with 18S rRNA and correspond to the average of a minimum of three experiments. Statistically significant differences among groups were evaluated by one-way ANOVA followed by Tukey´s post-test. Pairwise differences between treatment groups are indicated as * *p* < 0.05. Pairwise differences between each treatment group and control (untreated) condition are indicated as ## *p* < 0.01. ns = not significant. Doses of PIM/P/d (in nM): 100/250/2.5 for RPMI-8226; 200/500/5 for NCI-H929.


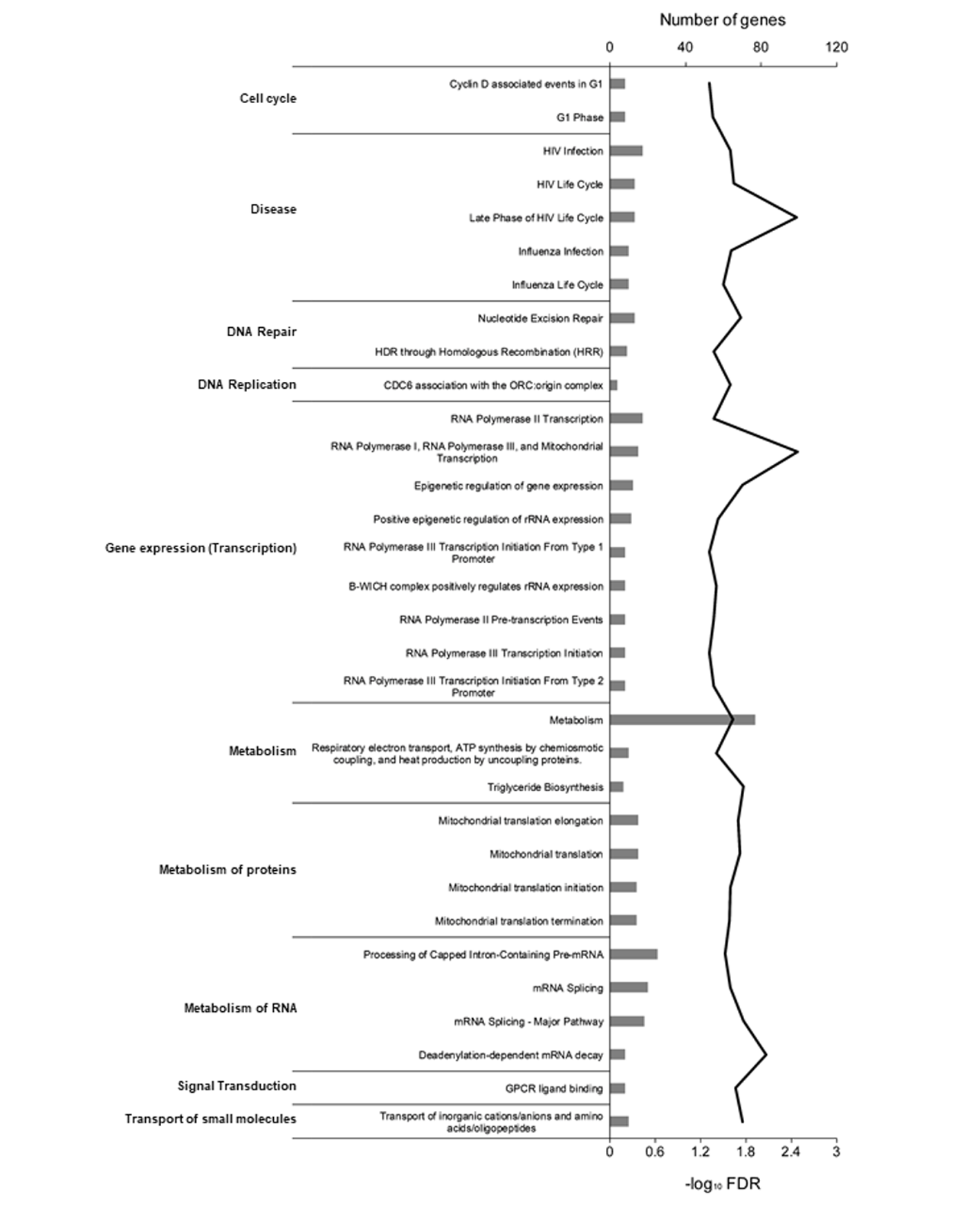


**Figure S13.** Pathways exclusively deregulated in MM.1S cells after treatment with PIM-Pd. The diagram shows the 32 exclusive pathways found to be significantly deregulated in a Gene Set Enrichment Analysis (GSEA) in MM.1S cells after treatment with PIM-Pd. The number of leading edge genes of each pathway and its statistical significance, expressed as −log10 FDR, are included. Top Level pathways, according to Reactome database, are indicated on the left side.


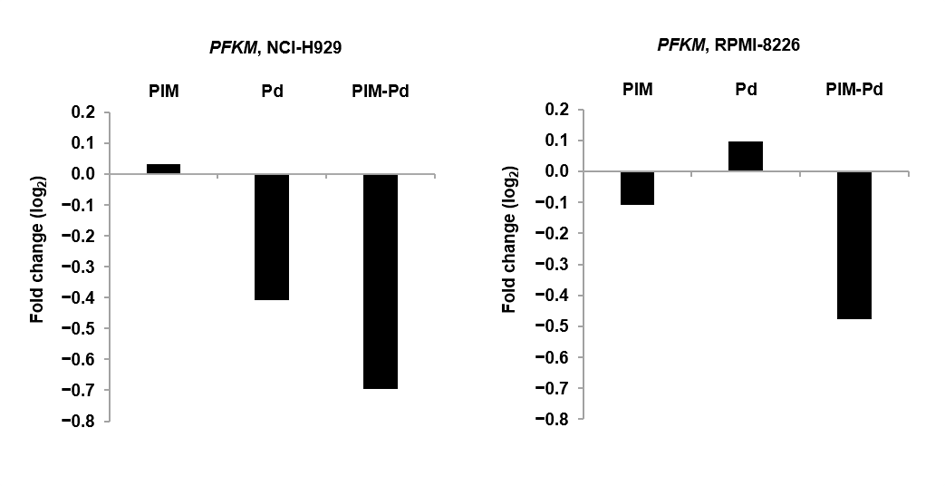


**Figure S14.** Effect of the triple combination PIM-Pd on the expression of *PFKM* in NCI-H929 and RPMI-8226 cell lines. NCI-H929 and RPMI-8226 cell lines were cultured in the absence or presence of PIM447 (PIM), pomalidomide + dexamethasone (Pd) or the triple combination PIM-Pd for 15 h. Subsequently, transcriptomic levels of *PFKM* were assessed by RT-qPCR. The results are shown as the magnitude of change between treated and untreated cells after normalization with 18S rRNA and correspond to the average of three experiments. Doses of PIM/P/d (in nM): 100/250/2.5 for RPMI-8226; 200/500/5 for NCI-H929.

| 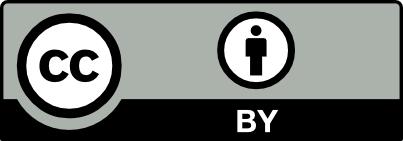 | © 2020 by the authors. Licensee MDPI, Basel, Switzerland. This article is an open access article distributed under the terms and conditions of the Creative Commons Attribution (CC BY) license (http://creativecommons.org/licenses/by/4.0/). |
| --- | --- |
