# Supplementary material for: Protein Translation Inhibition is Involved in the Activity of the Pan-PIM Kinase Inhibitor PIM447 in Combination with Pomalidomide-Dexamethasone in Multiple Myeloma
Source: Cancers (Basel). 2020 Sep 24;12(10):2743. doi: 10.3390/cancers12102743 (PMC7598606; doi:10.3390/cancers12102743)
Supplement: Supplementary file 1 [file cancers-12-02743-s001.zip › cancers-932840-uncropped Wb images.pdf]

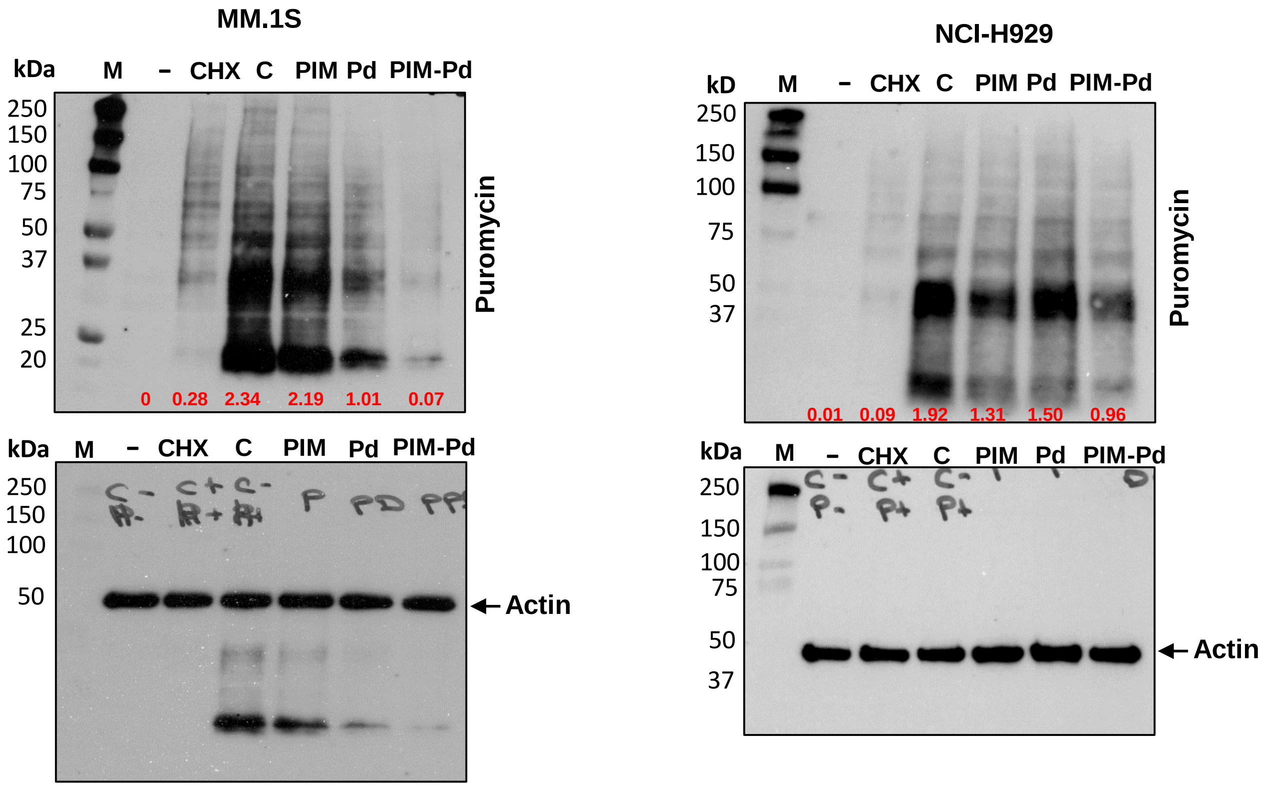

Western blot images supporting Figure 3A. M: molecular weight marker

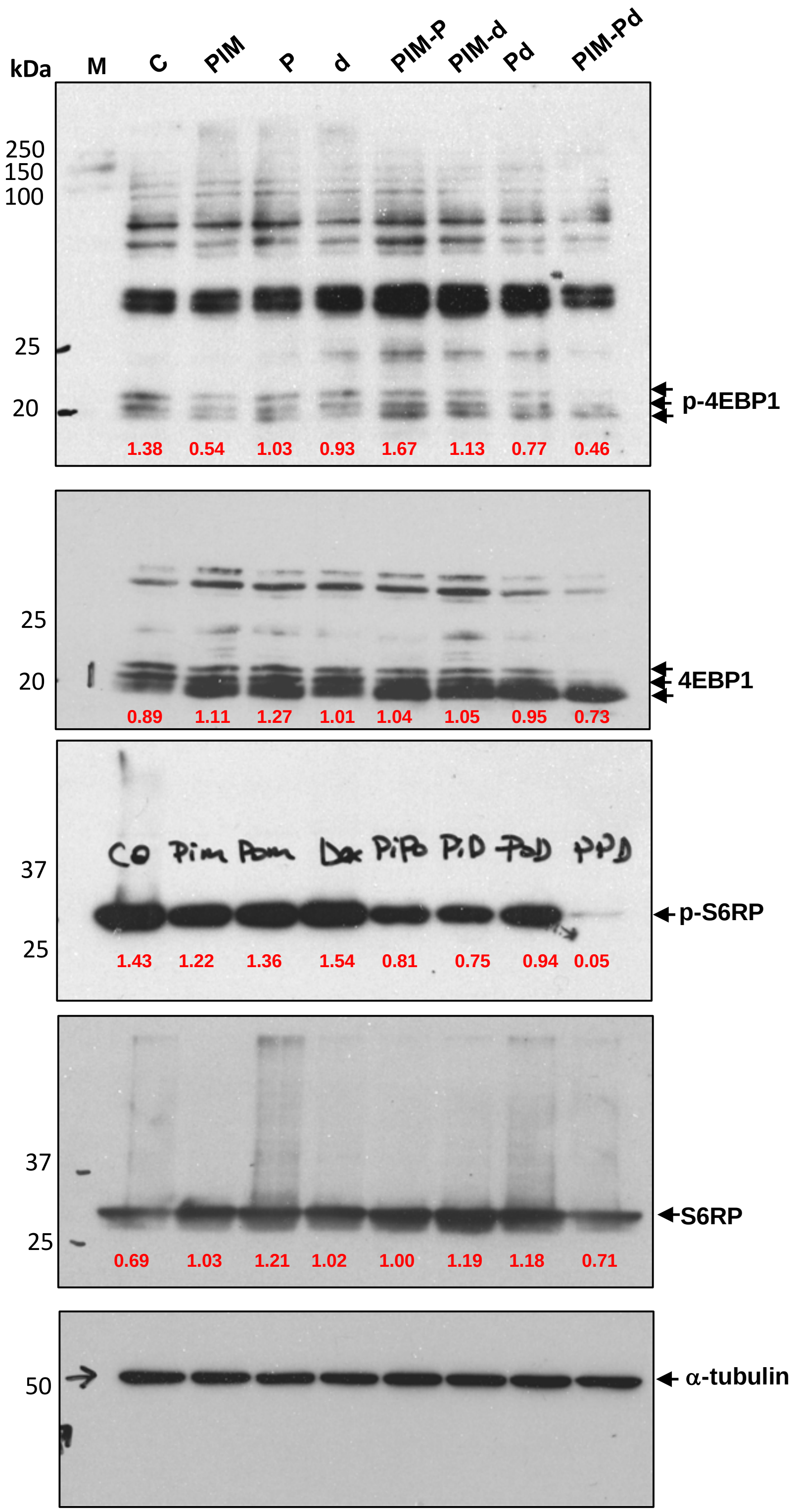

Western blot images supporting Figure 3B. M: molecular weight marker

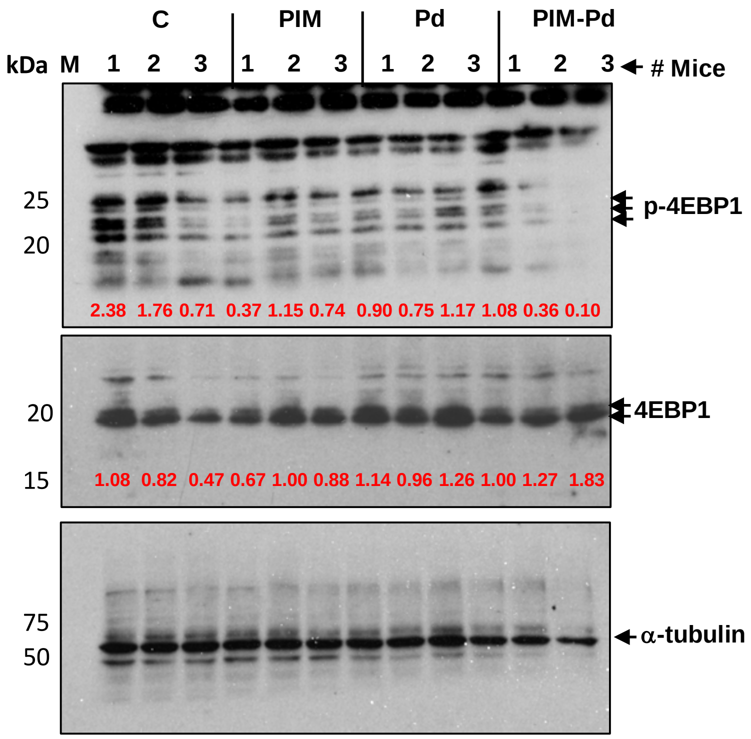

Western blot images supporting Figure 3C. M: molecular weight marker

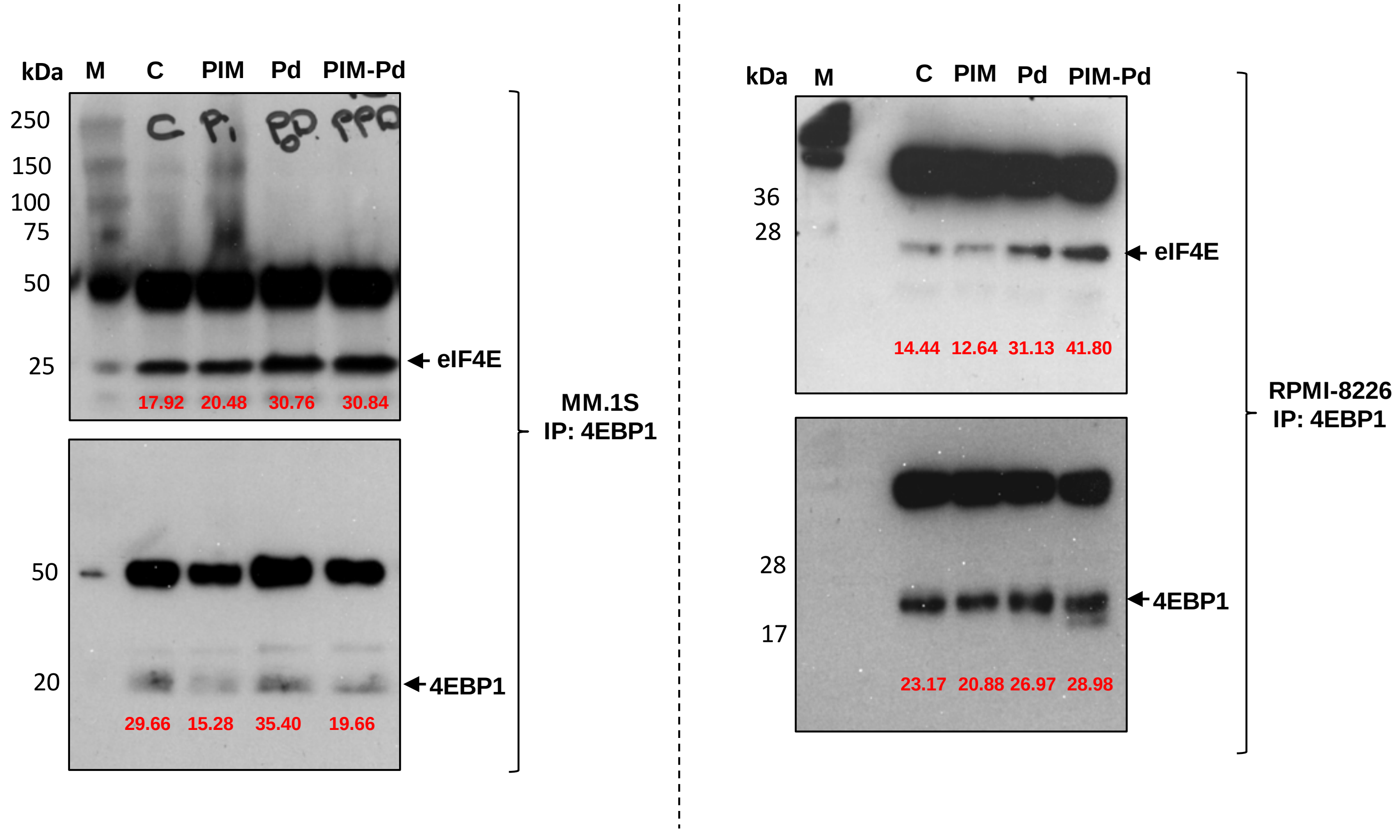

Western blot images supporting Figure 3D. M: molecular weight marker

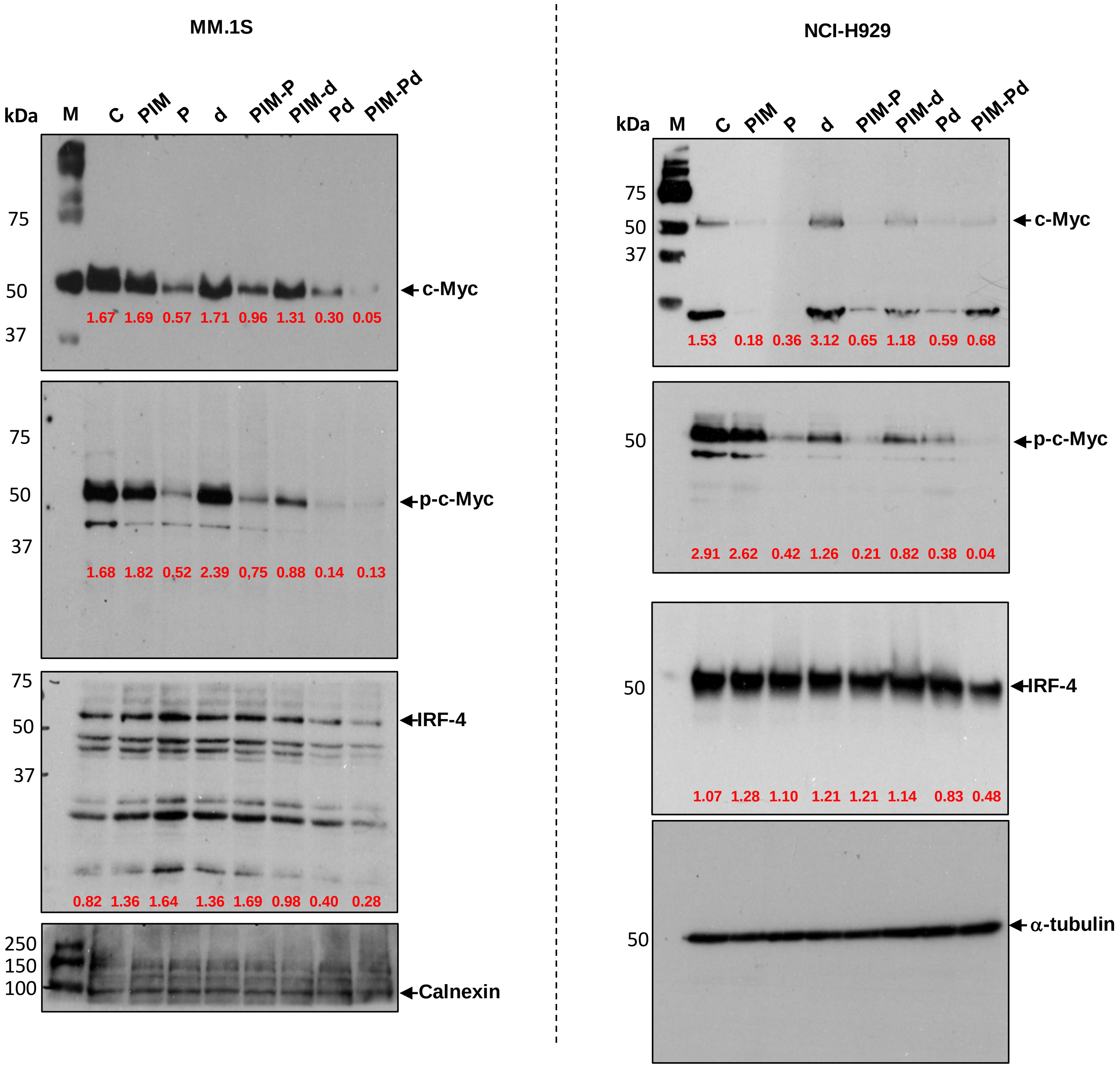

Western blot images supporting Figure 4A. M: molecular weight marker

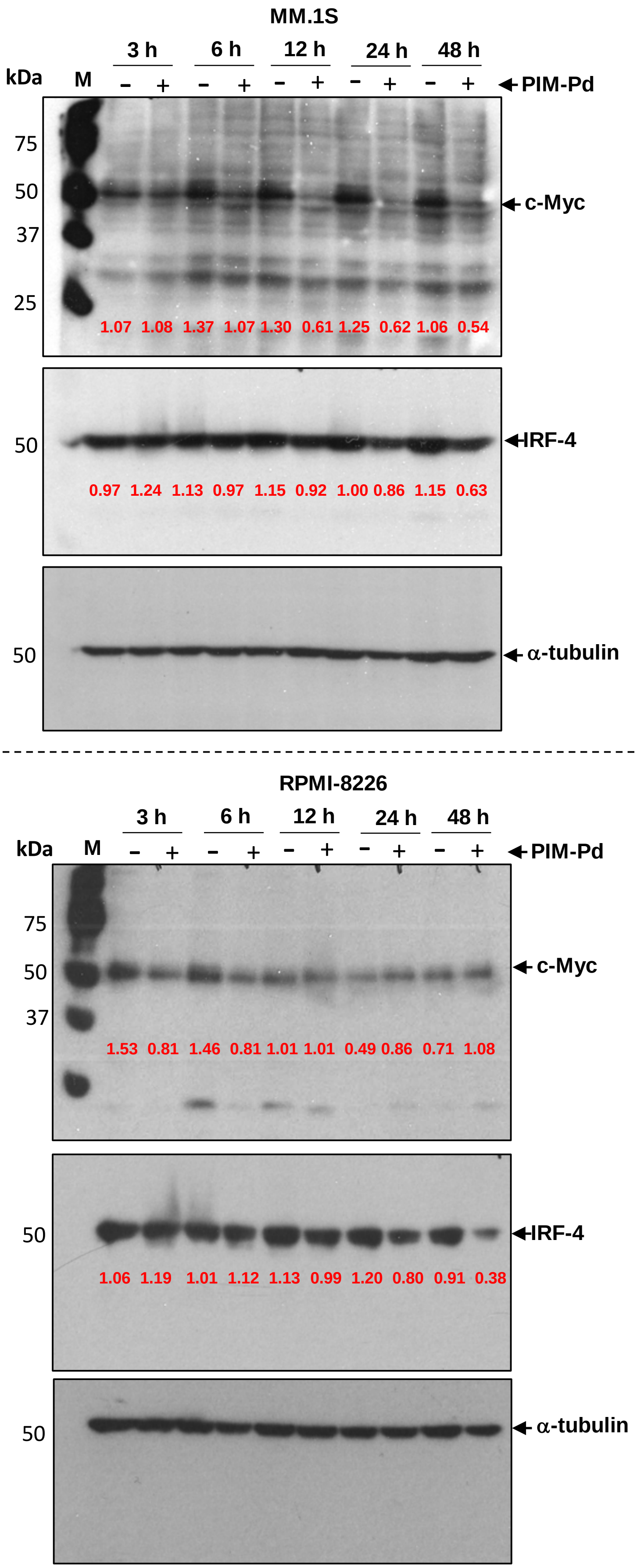

Western blot images supporting Figure 4C. M: molecular weight marker

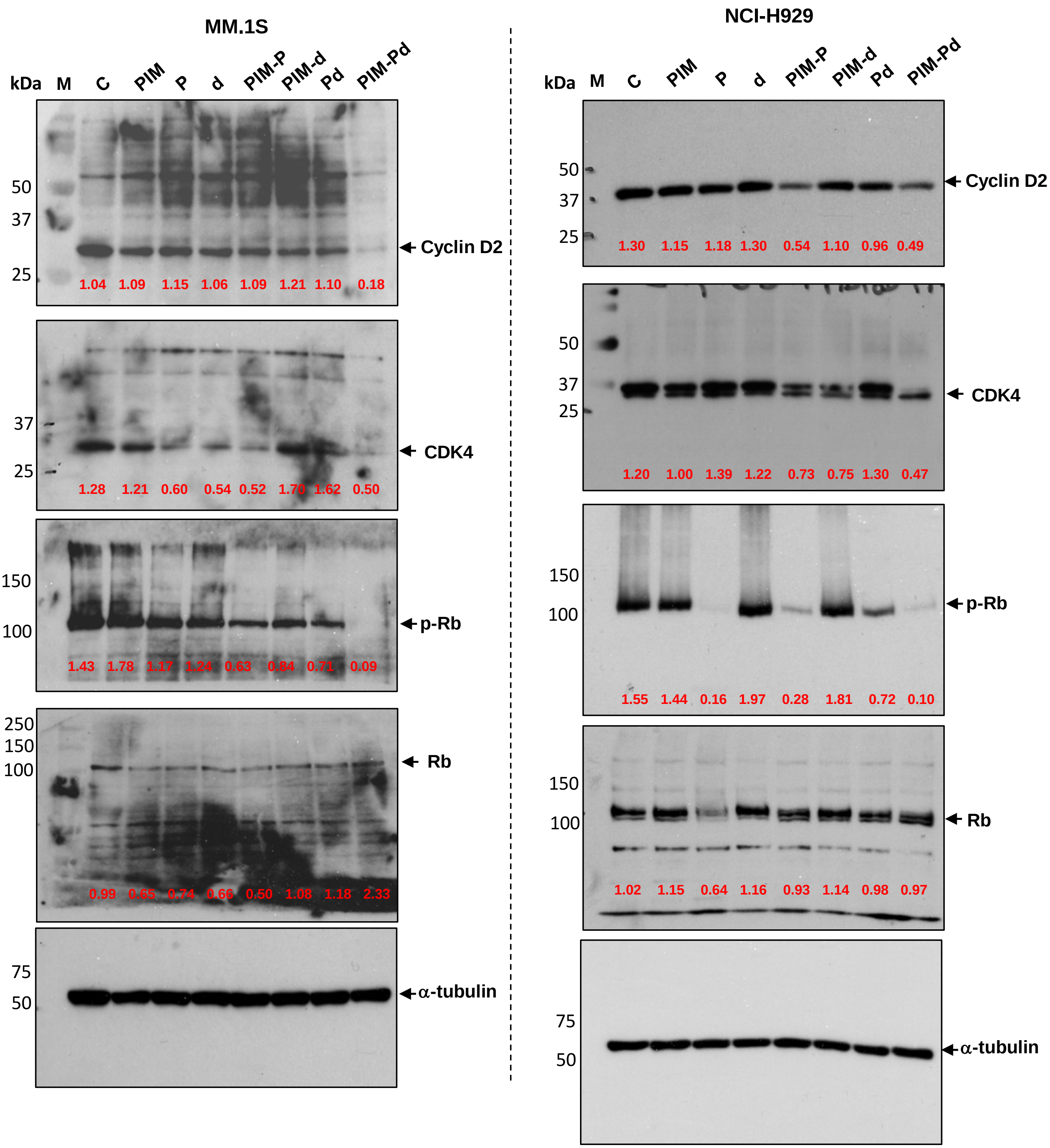

Western blot images supporting Figure 6B. M: molecular weight marker

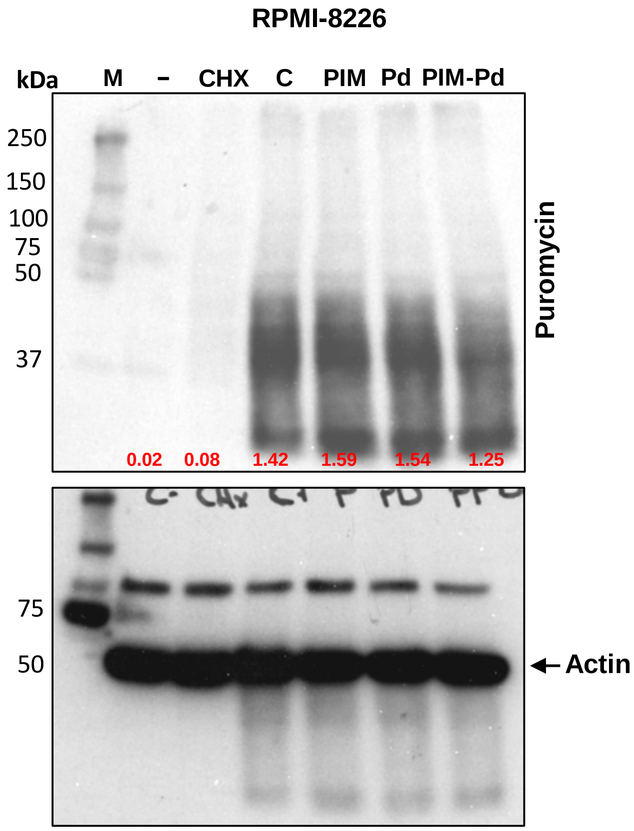

Western blot images supporting Figure S4A. M: molecular weight marker

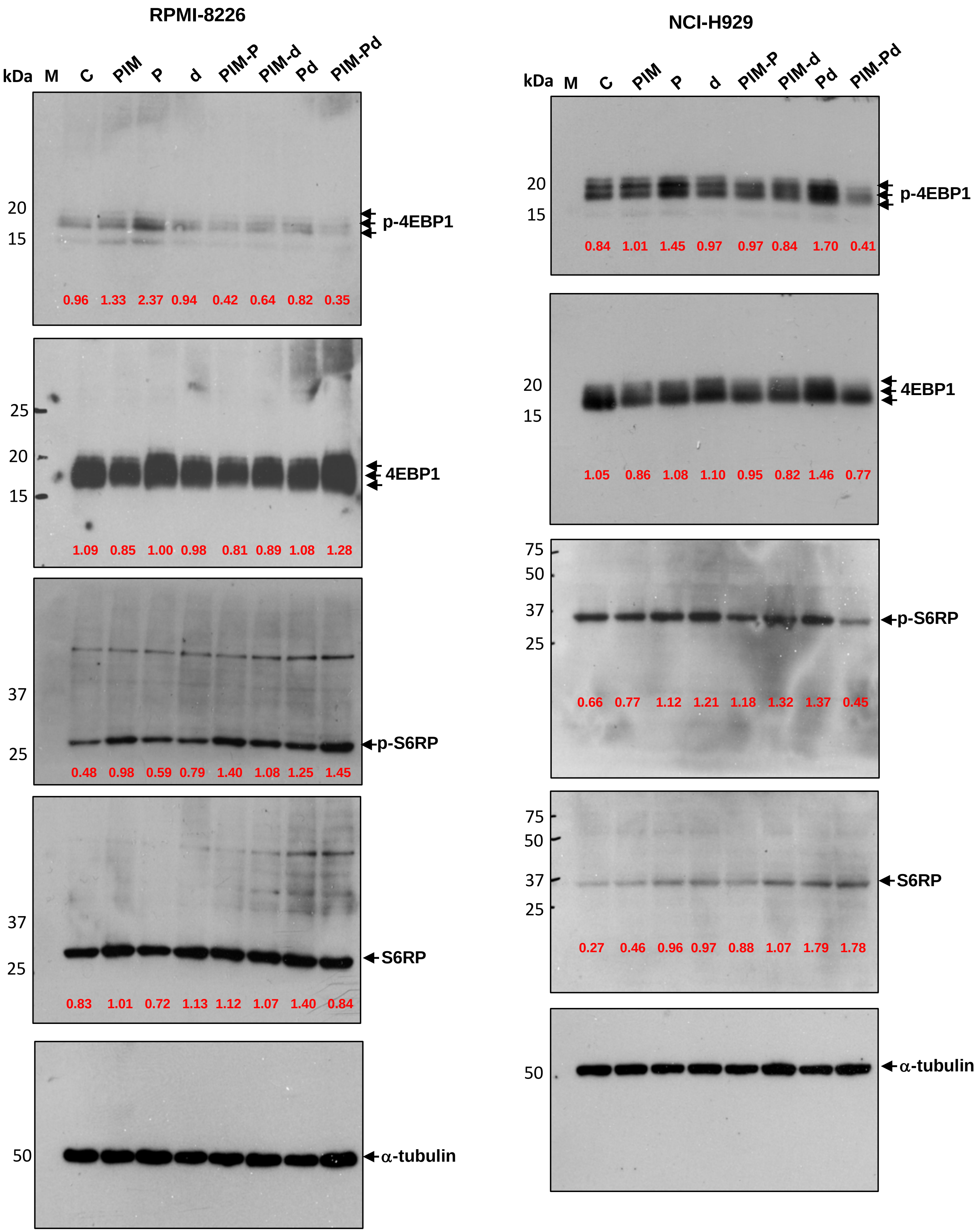

Western blot images supporting Figure S4B. M: molecular weight marker

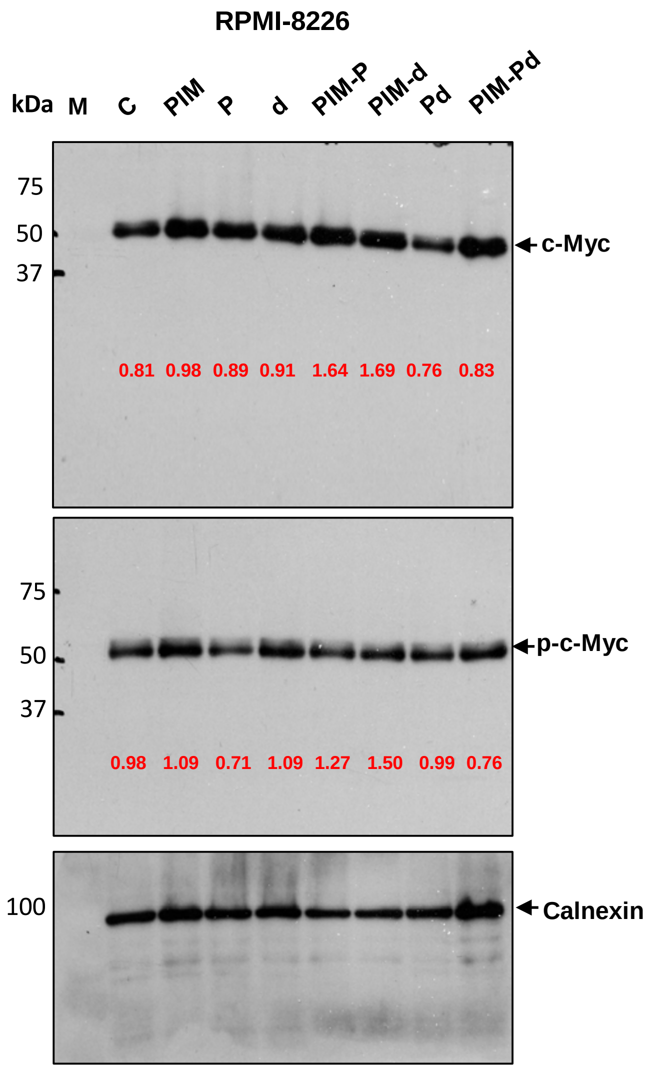

Western blot images supporting Figure S5A. M: molecular weight marker

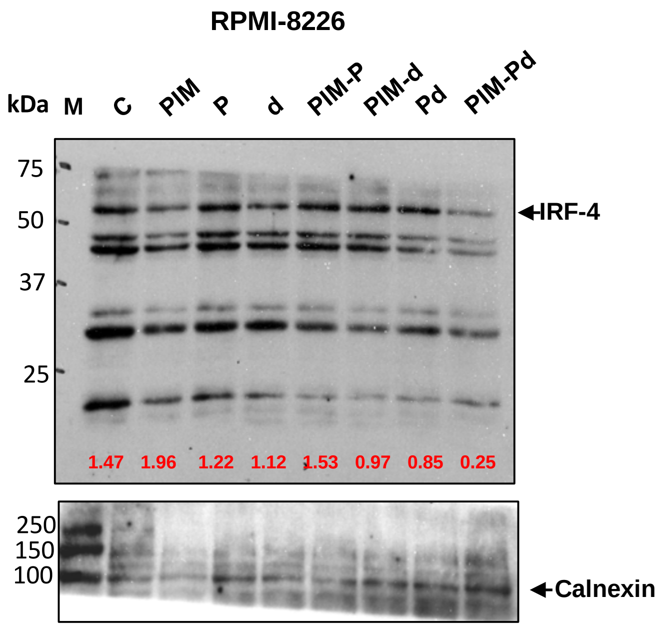

Western blot images supporting Figure S5B. M: molecular weight marker
